# Functional Characterisation of Alpha-Galactosidase A Mutations as a Basis for a New Classification System in Fabry Disease

**DOI:** 10.1371/journal.pgen.1003632

**Published:** 2013-08-01

**Authors:** Jan Lukas, Anne-Katrin Giese, Arseni Markoff, Ulrike Grittner, Ed Kolodny, Hermann Mascher, Karl J. Lackner, Wolfgang Meyer, Phillip Wree, Viatcheslav Saviouk, Arndt Rolfs

**Affiliations:** 1 Albrecht-Kossel-Institute for Neuroregeneration, Centre for Mental Health, University of Rostock, Rostock, Germany; 2 Institute of Medical Biochemistry and IZKF, University of Muenster, Muenster, Germany; 3 Department for Biostatistics and Clinical Epidemiology, Charité-University Medicine, Berlin, Germany; 4 Department of Neurology, New York University School of Medicine, New York, New York, United States of America; 5 pharm-analyt, Labor GmbH, Baden, Austria; 6 Institute for Clinical Chemistry and Laboratory Medicine, University of Mainz, Mainz, Germany; 7 Barts and the London School of Medicine and Dentistry, Queen Mary University of London, London, United Kingdom; 8 Institute for Molecular Diagnostics, Centogene GmbH, Rostock, Germany; University College London, United Kingdom

## Abstract

Fabry disease (FD) is an X-linked hereditary defect of glycosphingolipid storage caused by mutations in the gene encoding the lysosomal hydrolase α-galactosidase A (GLA, α-gal A). To date, over 400 mutations causing amino acid substitutions have been described. Most of these mutations are related to the classical Fabry phenotype. Generally in lysosomal storage disorders a reliable genotype/phenotype correlation is difficult to achieve, especially in FD with its X-linked mode of inheritance. In order to predict the metabolic consequence of a given mutation, we combined in vitro enzyme activity with in vivo biomarker data. Furthermore, we used the pharmacological chaperone (PC) 1-deoxygalactonojirimycin (DGJ) as a tool to analyse the influence of individual mutations on subcellular organelle-trafficking and stability. We analysed a significant number of mutations and correlated the obtained properties to the clinical manifestation related to the mutation in order to improve our knowledge of the identity of functional relevant amino acids. Additionally, we illustrate the consequences of different mutations on plasma lyso-globotriaosylsphingosine (lyso-Gb3) accumulation in the patients' plasma, a biomarker proven to reflect the impaired substrate clearance caused by specific mutations. The established system enables us to provide information for the clinical relevance of PC therapy for a given mutant. Finally, in order to generate reliable predictions of mutant GLA defects we compared the different data sets to reveal the most coherent system to reflect the clinical situation.

## Author Summary

Fabry disease is caused by a single gene deficiency. It is the second most common lysosomal storage disorder and the result is a build-up of glycosphingolipids in different areas of the body (kidneys, intestine, etc). It is an important consideration for clinicians in the diagnosing of stroke, kidney and cardiovascular diseases. Many symptoms of Fabry are seen in other diseases as well (both inherited and non- inherited), which makes diagnosis difficult. We observed numerous novel mutations in patients that displayed a monosymptomatic, however life-threatening course of Fabry disease. This prompted us to study and characterise those mutations with regard to their biochemical and clinical consequences. Overall, 171 Fabry mutations were considered in an overexpression system for the prediction of the clinical course of Fabry disease. Furthermore, we highlight the usefulness of the *in vitro* system that we developed which will help with therapeutical decisions, by testing the responsiveness of mutant enzymes to the pharmacological chaperone DGJ. This work aims to draw the attention of clinicians and researchers to milder forms of Fabry disease which might at first appear unrelated to this clinically heterogenous disease.

## Introduction

With a suspected prevalence of 1∶3,100 to 13,341 [1],[2], Fabry disease (FD, OMIM #301500) is the second most frequent lysosomal storage disorder. FD causes the accumulation of intracellular/lysosomal, plasma and urinary globotriaosylceramide (Gb3) which, due to mutations within the α-galactosidase A (*GLA*, Xq22) gene, cannot be cleared. Defects of the *GLA* gene product, caused mostly by single amino acid substitutions, lead to its early degradation within the endoplasmic reticulum [3] and prohibit intracellular trafficking of the enzyme to the destination organelle, the lysosome. In affected patients, typical FD presents as a multisystemic disorder and in classic cases stroke, acroparaesthesia, hypohidrosis, angiokeratoma, cornea verticillata, cardiac and kidney disease [4],[5] develop. However, milder mono- or oligosymptomatic cases have been reported [6]–[9]. Oligosymptomatic cases further impede the diagnosis, e.g., large numbers of abnormal variant forms of *GLA* found in newborn-screenings are either linked to a monosymptomatic and late onset of the disease [1],[2] or are coincidental findings that can be considered as variants without significant metabolic consequences. Some other mutations seem to be related to a certain phenotype with a predominantly single organ involvement, hence, both a cardiac variant [10]–[12] and a cerebrovascular variant [13] have been described. Typically these mutations are associated with a late onset phenotype. There appears to be at least one mutation (*p.D313Y*), most likely a polymorphism [14],[15] but which can also be found in stroke of unexplained aetiology [13],[16].

Due to the X-linked mode of inheritance, genetic sequencing in females is the only valid tool to diagnose FD, since enzyme activity in patient leucocytes can be in the normal range in a high number of female heterozygotes [17], even in those severely affected. Because of the X-linked inheritance, two thirds of all Fabry patients can be expected to be females.

Globotriaosylsphingosine (lyso-Gb3), a deacylated metabolite of Gb3 has been described as a useful biomarker to quantify the burden of FD. Lyso-Gb3 was shown to be the storage material in many cells accumulating to high levels in vasoendothelial cells of blood vessels. Lyso-Gb3 was proven to be more specifically increased in FD patients [18] than Gb3 and demonstrated to be reduced in mice under enzyme replacement therapy (ERT). Moreover, lyso-Gb3 has been proposed to take part in the development of FD nephropathy [19] and general inflammatory processes. Even though lyso-Gb3 can be used as a reliable biomarker for FD, it is only reliable for monitoring as no absolute correlation has been described between the nature of the mutation and lyso-Gb3 levels. It was concluded that long periods of exposure to this agent lead to the development of symptoms [20].

Several recent studies tried to generate more impartial data about the cellular and structural consequence of a given *GLA* mutation. They were typically based on overexpression systems and analysed either the residual GLA enzyme activity [21] or focussed on enzyme trafficking and stability aspects [3]. Other studies tried to establish genotype/phenotype correlations in clinical cases of Fabry patients [22]. The elucidation of the crystal structure of the enzyme [23] was the basis for the establishment of structure/function correlation models [24]–[26].

In a recent study, newly described mutations were analysed in an overexpression system. Enzyme activity was measured and responsiveness to 1-deoxygalactonojirimycin (DGJ) was tested in Western blot assay and in patient's T-lymphocytes [27]. This approach was used to determine the likelihood of a newly identified *GLA* variant leading to FD. Wider studies with T-lymphocytes have previously been undertaken [28],[29]. A systematic approach to examine a broad range of mutations has been developed in order to facilitate diagnosis and therapeutic decisions [30].

In the present study 171 *GLA* mutations were characterised biochemically for residual enzyme activity, degradation status and behaviour towards pharmacological chaperone (PC) DGJ. Moreover, the obtained data sets were compared with patient plasma lyso-Gb3 levels, the computational prediction algorithm PolyPhen2 (Polymorphism Phenotype v2) [31] and the clinical phenotype. We paid particular attention to mutations believed to be on the borderline for causing the disease.

## Results

### Determination of single amino acid substitution mediated α-galactosidase A damage

We tested 171 mutations with regard to enzyme activity and degradation status to evaluate which mutation leads to diminished enzyme activity. Among the mutations tested, 50 had not been described previously., In sum, 158 missense, 6 nonsense mutations causing immediate polypeptide abort, 4 small deletions (1–2 nucleotides) and 3 small insertions (1–4 nucleotides) were examined for potential damage (Table S1). Mutant enzymes were studied with respect to their effect on protein degradation and pharmacological chaperone responsiveness (Figure S1).

**Figure 1:**
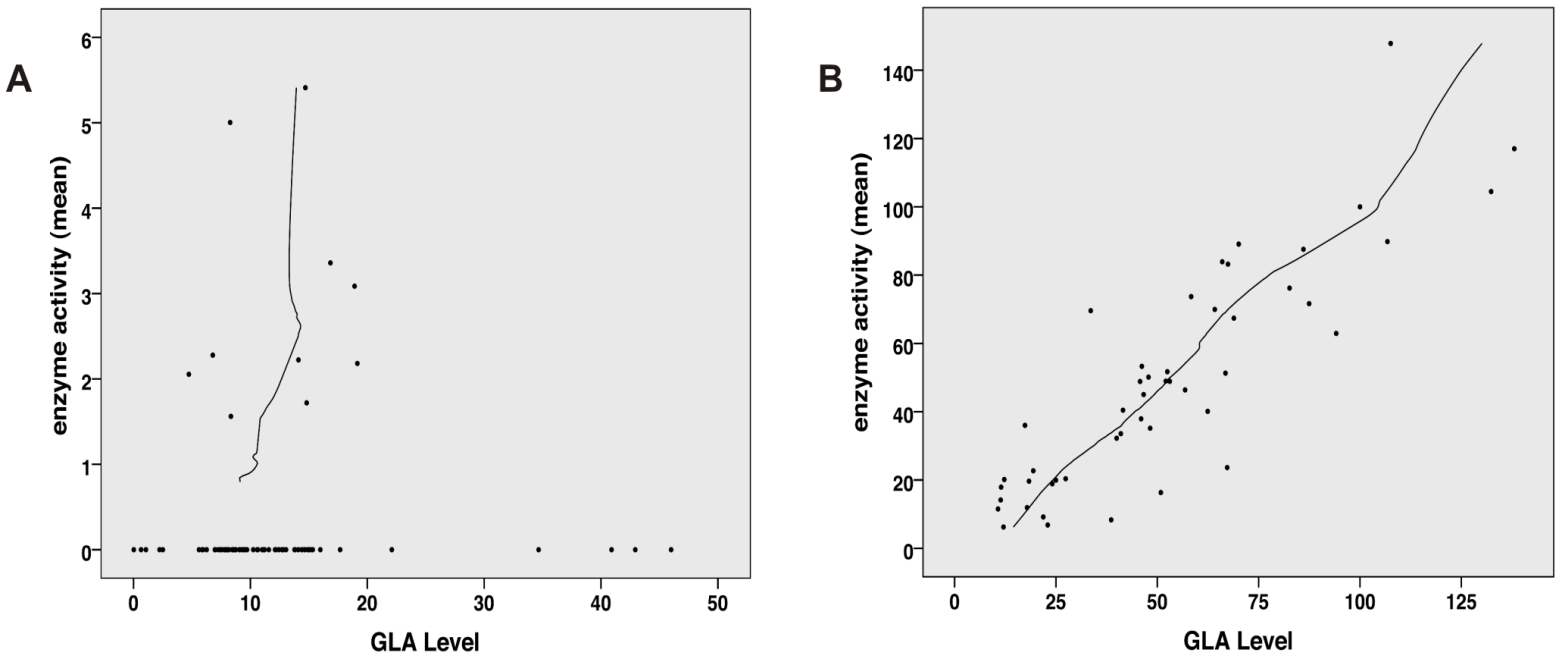
Correlation analysis of α-Gal A level (semi-quantitative Western Blot) and activity. **A:** No correlation between GLA level and residual activity for mutations possessing less than 6% residual activity (n = 76, Spearman correlation coefficient rs = 0.128, p = 0.272). This implies that the catalytic unit is affected by the mutation and thus high amount of enzyme cannot compensate for the loss of activity. **B:** For mutation possessing more than 6% residual GLA activity (n = 48), the *in vitro* enzyme activity and GLA levels correlate with each other, indicating that the catalytic core is still intact and mutation most likely affect protein trafficking (Spearman correlation coefficient rs = 0.866, p<0.001).

**Figure 2:**
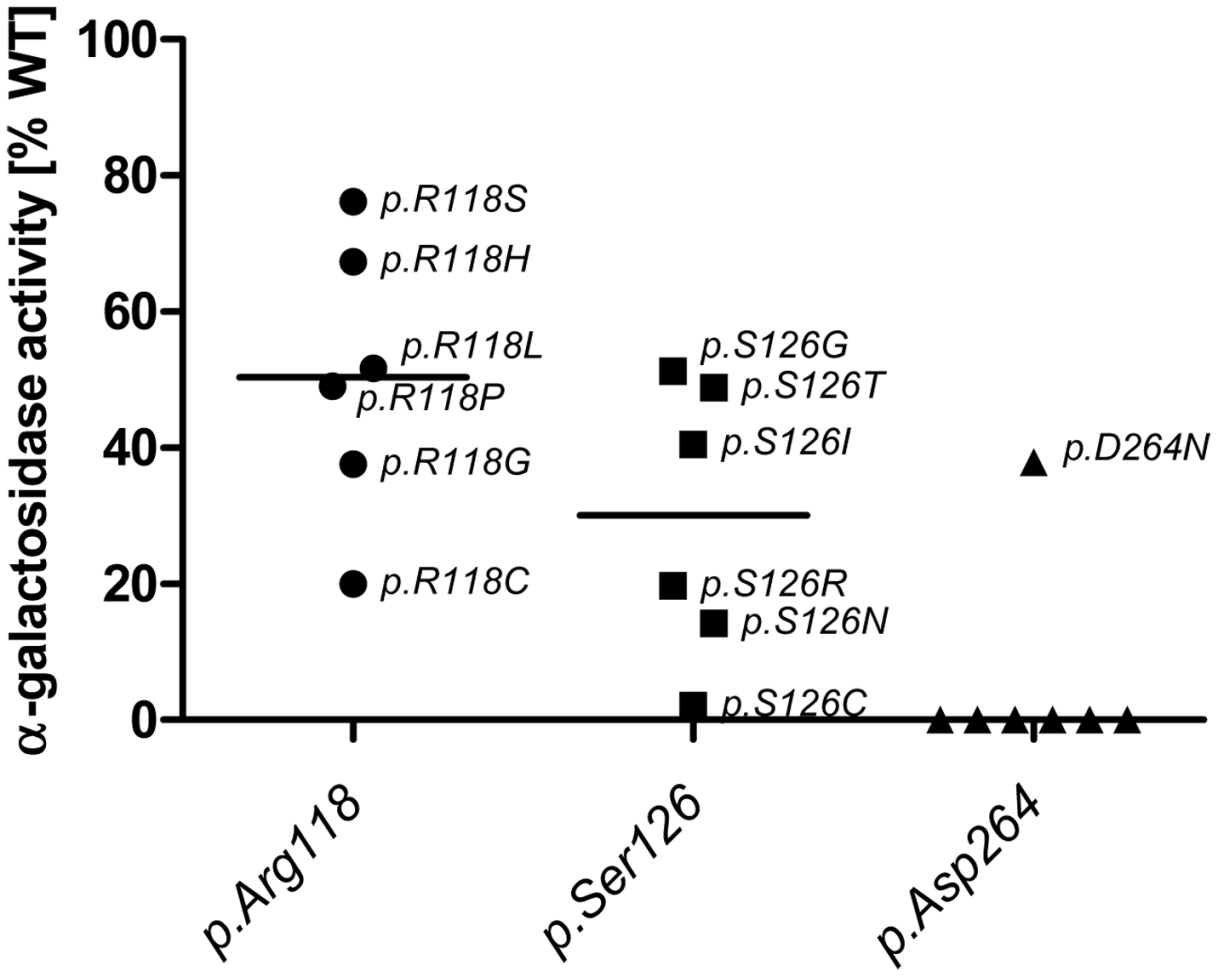
*In vitro* activity of specific GLA site mutations. Note that mutations at position *p.Asp264* almost always lead to a loss of GLA activity, while the same does not hold true for *p.Arg118* and *p.Ser126*. Interestingly mutations in *p.R118* do not lead to a loss of activity below 20% of WT and range from 20% to 80% while *p.Ser126* can lose all activity with certain mutations and retains no more than 60% activity. This highlights the differential effects of the mutational site and amino acid change on α-Gal A activity. Given is the median activity of all mutations in each position (horizontal mark).

Enzyme activity and intracellular enzyme level were correlated for mutations natively displaying activities above 6% (Spearman correlation coefficient rs = 0.866, p<0.001) (Figure 1B). Enzyme levels were not associated with residual activity since highly defective enzymes seemed to lack the capacity to process the substrate (Spearman correlation coefficient rs = 0.128, p = 0.272) (Figure 1A). Figure S1 shows enzyme content for some strongly affected enzymes (e.g. *p.D231N*, *p.L275F* and *p.L415P*) with no measurable activity. Overexpressed mutant α-Gal A was abundantly present in the cells, notably above the endogenous level at the expected size of 46 kDa. Thus, the protein was evidently processed [32], but it is not catalytically active. A closer look at position *p.Arg118* (buried residue) revealed that the actual described mutation for this moiety (*p.R118C*) displayed the lowest detected enzyme activity at about 20% of the wild type enzyme (Figure 2). All other mutations at this position (e.g. R118G, R118S, see Table S1) were less severely affected (range 20–70.9%). Vice versa, at position S126 (stabilising loop), the described mutation S126G displayed the mildest effect on activity loss. The strong decrease of activity for S126C is explained by the chemical nature of the amino acid exchange from serine to cysteine, predicted to disrupt protein conformation (due to auxiliary disulphide bond introduction). As expected when changing the important aspartate residue at position 264 (close active site) [33], the enzyme lost its activity. The only exception was *p.D264N* which maintained about 37.9% of its activity. We arranged 4 classes of mutations based upon the enzyme activity data: class I: 0%, class II: >0%–20%, class III: ≥20%–60%, class IV: ≥60% for further analyses.

### Responsiveness to pharmacological chaperone DGJ

In order to examine the importance of enzyme activity for clinical and therapeutical decisions, we analysed enzyme activity changes after addition of the pharmacological chaperone DGJ. In order to simplify the outcome measures, a responder was defined as a mutation whose activity was increased 1.5-fold or >5% compared to the untreated value. Under this directive, 42.8% of the missense mutants were responsive to DGJ. Mutations in sites *p.Arg118*, *p.Ser126* and *p.Asp264* gave rise to enzymes that displayed an overall minor (if any) increase of activity through DGJ treatment indicating that the moieties are generally situated in sites where treatment with the PC does not result in significant elevation of α-Gal A activity and that the type of amino acid substitution is biochemically less relevant (exception: *p.D264N*, see also Table S1). Furthermore, DGJ analysis showed that active-site-associated amino acid substitutions (*p.D93E/Y*, *p.D170N*, *p.R227Q* and *p.D231N*) could not retain lost activity under treatment.

DGJ responsiveness was highly associated with residual activity of the mutant enzyme *in vitro* (p<0.001 in linear trend test, Table S2). Class I mutants were less likely to respond to PC treatment (14.5%) than class II mutants (82.8%) with a less severe impact on enzyme function. Most of the best responders belong to this category (*p.A156V*, *p.I253S*, *p.R301Q*, etc.). In classes III and IV, a high percentage of responders was present (71.4 and 63.2% respectively), though with a high number of only weakly stimulated enzymes.

### Association of different parameters

In a first approach, we compared residual enzyme activity to biomarker levels obtained from patients with the according mutations to investigate whether *in vitro* data reflect lyso-Gb3 as a marker for clinical severity.

**Figure 3:**
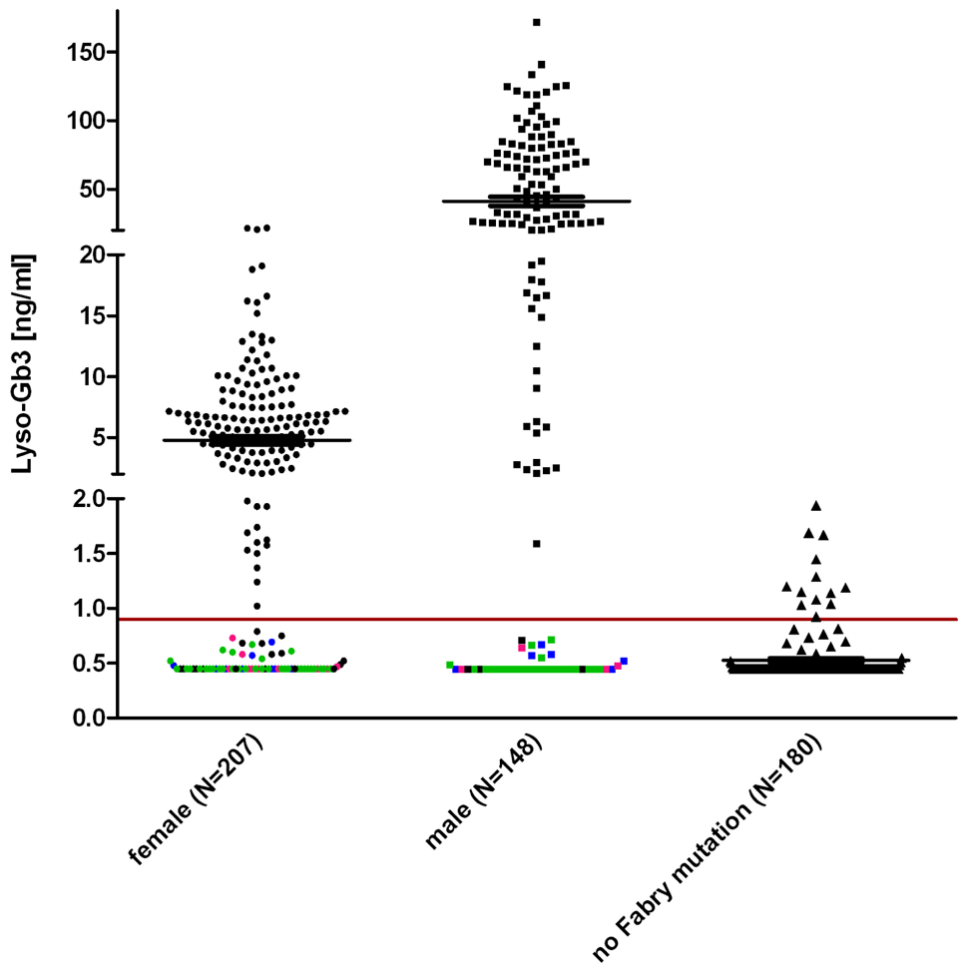
Lyso-Gb3 values for female and male Fabry patients compared to control. The horizontal mark indicated the median. It is noteworthy that lyso-Gb3 levels in males are ∼10 times higher than in females. Each data point represents one patient. Indicated in pink are patients with the mutation *p.S126G* (8f/4m), in blue *p.A143T* (10f/8m) and in green *p.D313Y* (33f/24m) to illustrate that most found non-pathogenic mutations belong to either one or the other patient cohort. Other exceptions are: *p.R118C*, *p.V316I*, *p.E418G* (one male patient each) and *p.A20P*, *p.D83N*, *p.I91T*, *p.S102L*, *p.R112C*, *p.R118C*, *p.D175E*, *p.G325S*, *p.A368T*, *p.T385A*, p.W399*, c.1208delT, *p.L415F*, (one female patient each) and *p.R252T* (4×), *p.N215S* (3×). About 180 healthy probands were tested with no Fabry gene variation and had values of 0.9 ng/ml (95^th^ percentile calculation).

To make sure that lyso-Gb3 was an adequate FD measure for testing of *in vitro* enzyme activity accuracy, we ascertained that lyso-Gb3 in male and female Fabry patients (Figure 3) were considerably higher than lyso-Gb3 in healthy controls [34]. The pathological cut-off for lyso-Gb3 measurements was set to 0.9 ng/ml (95th percentile of healthy individuals).

The gender-dependence of lyso-Gb3 values in hemizygote males and heterozygote females carrying the same mutation (compare Table S1) reflects the shortcoming of patient-derived data. Still, the median values of the patient/proband cohorts showed values above the pathological cut-off in both males and females (5.5 ng/ml for female individuals, 28.6 ng/ml for males). In figure 3 coloured dots represent patients with the mutations *p.S126G*, *p.A143T* and *p.D313Y*, including most of the values not detected as pathological. Strikingly, the overall inconspicuousness of mutations such as *p.A143T* or *p.D313Y* (see mean values in Table S1) was not gender-dependent, meaning that those patients would remain undetected in males and females alike. However, Table S3 shows that lyso-Gb3 displays sensitivity for classic mutations. Depicted is another set of mutations found in patients where we measured plasma lyso-Gb3. In this table, most of the mutations led to a complete loss of the enzyme (truncations, splice mutations, etc). The positive predictive value for lyso-Gb3 (for all 355 FD patients and 180 control individuals, Figure 3) was 95%. The negative predictive value was 60.9%.

Female and male biomarker data were analysed separately to evaluate the association between enzyme activity and levels of the biomarker lyso-Gb3. For the categorisation of lyso-gb3-values we used the following cut-points: class I: lyso-gb3 ≥80/10 ng/ml for males/females, class II: lyso-gb3 20–80/5–10 ng/ml for males/females, class III: lyso-gb3 0.9–20/0.9–5 ng/ml for males/females, class IV: lyso-gb3<0.9 ng/ml (for males/females). The enzyme classes were strongly associated with the biomarker levels of the patients (p<0.001 for males and for females, linear trend test, Table 1). Mutations with an activity lower than 20% showed gender-independent elevated lyso-Gb3 values.

**Table 1:** Association of *in vitro* enzyme activity and clinical and computational parameters.

	enzyme activity				
	0%	>0%–20%	≥20%–60%	≥60%	p (for linear trend test)
**disease phenotype**					
classic	42	8	2	0	<0.001
classic/variant	3	1	3	0	
variant	1	2	6	4	
**lyso-gb3 male**					
I (≥80 ng/ml)	9	0	0	1	<0.001
II (20–80 ng/ml)	16	3	0	0	
III (0.9–20 ng/ml	4	2	3	1	
IV(<0.9 ng/ml)	0	0	3	3	
**lyso-gb 3 female**					
I (≥10 ng/ml)	5	0	0	0	<0.001
II (5–10 ng/ml)	13	1	0	0	
III (0.9–5 ng/ml)	9	4	4	1	
IV (<0.9 ng/ml)	0	0	4	7	
**PolyPhen2**					
benign	5	2	12	13	<0.001
possibly damaging	8	7	4	1	
probably damaging	68	14	11	2	

Statistical association of enzyme activity and other parameters indicative for Fabry disease. The matrix confirms the high degree of translatability of *in vitro* data to the individual patients' biomarker phenotype. Lyso-Gb3-based classes of the mutants are fitting the enzyme activity classes.

Trend test analysis revealed statistically significant associations with enzyme activity (p<0.001, linear trend test, Table 1). The *in silico* prediction tool PolyPhen2 revealed strong association with the enzyme activity classes as well (p<0.001)

In addition, we also considered the correlation of *in vitro* enzyme activity with accessible surface area of the mutated amino acid residues as a possible determinant for the biological consequence of a given mutation as proposed previously [33]. However, we found only a weak linear trend (for details see Table S2).

### Association of enzyme activity reduction to clinical phenotype

Table 2 shows the proportion of mutations correctly classified into the clinical phenotype groups by using lyso-Gb3 values of females, males, *in vitro* enzyme activity and PolyPhen2 scores. Enzyme activity showed the highest rates of correct classification both for 72 mutations where data were available and for 21 mutations for which we had values in all four measures (PolyPhen2 scores, lyso-Gb3 for females, lyso-Gb 3 for males and enzyme activity). The classification rates were 83% for 72 mutations and 86% for the subset of 21 mutations. Lyso-Gb3 for males and PolyPhen2 scores showed similar prediction quality: PolyPhen2 scores led to a slightly higher classification rate of 76% in the subset compared to 71% for lyso-Gb3 values for males. With 67% in the subset of mutations lyso-Gb3 values for females showed the lowest rate of correctly classified mutations.

**Table 2:** *In vitro* enzyme activity reflects clinical phenotype and is a predictor for FD.

	Lyso-Gb3 for females	Lyso-Gb3 for males	*In vitro* enzyme activity	PolyPhen2 classes
	N = 33	N = 32	N = 72	N = 72
Nagelkerkes R^2^	0.44	0.41	0.53	0.12
−2 Log Likelihood (df)	12.44 (3)	14.43 (3)	15.41 (3)	16.13 (2)
Mutations correct classified	69.7%	78.1%	83.3%	73.6%
	N = 21	N = 21	N = 21	N = 21
Nagelkerkes R^2^	0.45	0.40	0.82	0.53
−2 Log Likelihood (df)	12.91 (3)	13.44 (3)	4.18 (3)	7.80 (2)
Mutations correct classified	66.7%	71.4%	85.7%	76.2%

Comparison of prediction quality for different parameters with regard to clinical phenotype (classic, variant, classic/variant, see Table S1); variance estimates from ordinal regressions for the outcome ‘clinical phenotype’ with different covariates, 1. for all data available in the particular dimension, 2. for a subset of 21 mutations that have values in all dimensions.

## Discussion

### The validity of potential biomarker lyso-Gb3

Until now, biomarker data for novel mutations have been regarded as the gold standard for diagnosis (5) immediately following genetic diagnosis. This may not be accurate for milder cases of the disease, for example when the patient is tested early in the development of the disease (even though an age dependent increase is still speculative) or the mutation leads to a minor catalytic defect, since we failed to detect our *p.S126G*, *p.A143T* and *p.D313Y* patients (mean values are non-pathological, see Table S1) as well as three (female) *p.N215S* cases. This is in accordance with recent findings [35]. We reported a family with only female mutation carriers [36]. In the case of an unknown mutation this is a difficult situation, because lyso-Gb3 analysis is less strongly associated with disease phenotype in females (Table 2). For some newly described mutations, we lacked a detailed clinical description, however the phenotype and hence rationale for FD testing was either stroke (e.g. *p.D83N*, *p.S102L*, *p.N139S*, *p.R252T*, *p.V316I*, *p.L415F*, *p.E418G*), kidney disease (e.g. *p.A37T*, *p.H225D*, *p.E398A*) or in one case cardiological symptoms (*p.R220Q*) of unexplained etiology. A surprisingly high number of these missense mutations was found in oligosymptomatic patients and in the event of low lyso-Gb3 and high residual activity values. About 7.1% of males and 16.7% of females lyso-Gb3 (mean) of all mutations examined (Table S1) were within the non-pathogenic range. However, if we subtract all mutations related to mono- or oligosymptomatic FD, lyso-Gb3 is pathogenic for 100% of males and 96.9% of females. The remaining 3.1% of females harbour the following mutations: *p.A20P*, *p.I91T*, p.W262* and p.W399*. The truncating mutation p.W262* has not been described before but is expected to cause classic FD. The biomarker lyso-Gb3 demonstrated a weaker association to clinical phenotype than *in vitro* enzyme activity for each mutation studied. Table S3 shows additional 52 mutations for which biomarker data was collected. All classical mutations showed elevated values above the normal range in males.

For each mutation studied mean lyso-Gb3 values were always higher in male than in female patients (higher sensitivity in males). However the values obtained in females also accurately reflect disease pathology (similar specificity). It has to be emphasised that every mutation causing a pathological lyso-Gb3 mean value in males also demonstrates with a pathologically elevated mean value in females. In the same vein, the mutation N215S that has a higher frequency in the population manifests with a mean value of 4.2 ng/ml (male) obtained from 7 different patients displaying increased lyso-Gb3 and 1.1 ng/ml (female) obtained from 6 different patients. However, 3 of the females showed normal values of 0.79 ng/ml, 0.68 ng/ml, one individual had a value below the limit of quantitation (<LLOQ), respectively. Essentially, lyso-Gb3 is a marker of classic rather than uncertain FD, (i.e. monosymptomatic or mild cases) reflecting disease severity after all. However, to overcome the limitation of individual patient constitution, we propose a new classification system based on *in vitro* enzyme activity.

### In vitro enzyme activity as a tool for diagnostic management

Previous studies have focused on mutant enzyme activity, but this has never been proposed for use as a diagnostic tool. In the present study we utilised a cell culture model to generate enzymatic data for a large subset of *GLA* mutations. As stated before, Fabry disease-causing mutations are not limited to active site residues [37], but rather distributed across the entire protein. We therefore chose mutations in domains across the entire protein (see also criteria in the Materials and Methods section). Herein, we report and characterise 171 mutations on the basis of *in vitro* overexpression and enzyme activity. Among the tested mutations, 50 (29.2%) had not been described previously (note: the *p.Arg118*, *p.Ser126* and *p.Asp264* substitutions had not been found in patients yet, see Table S1). A strong association to biomarker plasma lyso-Gb3, the PolyPhen2 *in silico* prediction and the DGJ responsiveness of the mutation identifies those parameters as potential predictors of the clinical phenotype of the mutations. However, *in vitro* enzyme activity measurement was the most accurate predictor of disease phenotype assigning 85.7% of the mutants to the correct disease group whereas biomarker lyso-Gb3 for females and males and PolyPhen2 analysis only predicted 66.7, 71.4 and 76.2%, respectively, (as indicated by the highest explained variance in regression (R^2^), lowest −2Log-Likelihood, and highest rate of correct classified mutations, Table 2). The ability of the mutations to respond to PC treatment classified 76.2% of the mutation correctly (data not shown).

The predictive quality of PolyPhen2 is hampered especially when it comes to mutations that are in unknown molecular interaction sites or mutations in domains with unclear structural significance, such as the N-terminal signal peptide region (i.e. *p.A15E* is incorrectly predicted to display a benign impact on enzyme function). However, the precision could assumingly be enhanced using a specifically constructed algorithm as has been conducted for the prediction of DGJ responding mutants in FD [26]. The pharmacological chaperone DGJ can rescue unstable, degradation-prone mutants, therefore responsiveness to DGJ could be correlated with the level of damage of the mutation as proposed previously [26]. Since only 14.5% of class I mutants could be rescued with the pharmacological chaperone DGJ and the proportion of responding mutations is much higher within the other classes, residual activity is associated with whether a mutation can be rescued or not (p<0.001, Table 1). As mentioned before, the drawback for the lyso-Gb3 is reasoned by clinical variability of the individual patients, especially in females.

### Unknown and mild GLA mutations - the challenge to establish pathogenicity

Due to the fact that genotype/phenotype correlations only exist for severe *GLA* mutations and are mostly linked to the classic phenotype, clinical decision-making in diagnosis and therapy in Fabry disease is challenging. Further, the genotype/phenotype correlation depends on the patients' gender. However, there is no doubt that clinicians would apply an ERT to a female patient with a classic FD mutation as well as to males in pre-clinical or milder forms of the disease, since therapeutic success is closely connected to the correct treatment schedule and a timely onset of the treatment to improve the probability of a complete symptom reversal. Before the year 2000, *p.D313Y* had been described as the only enzyme variant with high residual activity and was therefore classified as a SNP. In contrast *p.A143T* which also has significant residual activity [28],[38], was classified as a disease-causing mutation [39] and also appears more frequent than usual FD mutations. The number of mutations resulting in a milder disease phenotype increased in the course of systematic screening programs identifying mutations in patient cohorts with symptoms common for other diseases. This highlights the need to re-consider the approach to Fabry diagnosis.

### Mutations with residual activity undergo early degradation

Due to the fact that FD mutations can affect both the stability and catalytic function of the enzyme we correlated the level and activity of GLA enzyme (Figure S1, Figure 1). The mutations *p.D93E* and *p.D231N* had GLA levels of 8.0% and 46.0% of wild type respectively, but no residual activity. Both mutants responded to the stabilising effect of DGJ, increasing GLA levels to 24.7% and 66.7% respectively, but no increase in activity was detected (Figure S1). In contrast to this finding class III and IV mutations as well as milder affecting class II mutations (activity >6%) displayed a strong correlation between GLA level and activity (Spearman correlation coefficient rs = 0.866, p<0.001). This emphasises the assumption that class III–IV (and the majority of class II) mutations mainly demonstrate an early degradation defect that retains enzyme activity. To test if mutations can affect stability alone, we performed a kinetic assay. Many of the GLA variants (*p.A143T*, *p.A156V*, *p.R301Q*, *p.L310F*) had unchanged kinetic properties (Table S4) indicating that the main reason for the molecular defect is early degradation. At least 3-fold higher activity above endogenous GLA was necessary to calculate kinetic parameters. Some of the mutations failed this criterion (*p.R49G*, *p.S65I*, *p.D231N* and *p.L415P* had activities around 200 nmol 4-MU/mg protein/hr which corresponds to endogenous GLA) and were therefore excluded from the analysis. However, the finding that even large amounts of enzyme could not increase activity above the endogenous level of the HEK293H cells indicates a severe impairment of the kinetic properties. Furthermore, this analysis revealed one mutation provoking only a mild decrease in enzyme activity, strong DGJ responsiveness and a benign *in silico* prediction that might nevertheless significantly change the kinetic properties of the enzyme (*p.H46P*). This could explain the disease pathology in patients with this mutation, since an increased K_M_ value could lead to diminished intra-lysosomal substrate turnover. Evidence rises that classification of mutants as either active site mutations or enzyme stability-abolishing mutations may not explain all aspects of the underlying defect. This was further explored by analysing a possible impact on the splicing behaviour resulting from the respective mutation. Supplementary Tables S5 A and B report on a few variants where the effect on splicing at exon-intron junctions as well as on cryptic splicing sites may contribute to their pathogenicity (for method see Text S1 in the supplementary material). However, the majority of missense mutations exhibit no noticeable effect on splicing.

Altogether, we propose to conduct all available testing (i.e. lyso-Gb3, *in silico* and *in vitro* overexpression analysis) to determine the characteristics of novel *GLA* mutations even though in severe cases it might be sufficient to test just one of the discussed parameters, but detailed testing might reveal unforeseen damage to the enzyme and its function. We point out the usefulness of a readily available *in vitro* assay in deciding whether an ERT or alternative treatment strategies (DGJ is currently being tested in clinical trials) should be started and whether a patient should be treated after pre-symptomatic genetic diagnosis at all, showing increased FD risk (harbouring a mutation with residual activity lower 20%) or if a frequent follow-up on the patient is initially more reasonable. However, only epidemiologic studies could determine to which extent mutations with a substantially lowered, yet high, enzyme activity (e.g. *p.D83N*, *p.S126G*, *p.A143T*, *p.D313Y*) contribute to the symptoms of Fabry disease and what role the degree of variation plays among individuals.

## Materials and Methods

### Patients and blood samples

Blood samples were obtained from patients undergoing biochemical analysis or genetic testing for verification of Fabry disease by the Albrecht-Kossel-Institute for Neuroregeneration (AKos). All patients agreed for testing of their blood samples. The project was in concordance with the regulations of the local Ethical Committee of the University Rostock.

### Cell culture

HEK293H cells were maintained in DMEM (Dulbecco's Modified Eagle Medium, Invitrogen, Karlsruhe, Germany) supplemented with 10% FBS (fetal bovine serum; PAA Laboratories, Pasching, Austria) and 1% penicillin/streptomycin (Invitrogen, Karlsruhe, Germany). All cells were incubated in a water-jacket incubator (Binder, Tuttlingen, Germany) at 37°C under 5% CO_2_ atmosphere. DGJ (Sigma Aldrich, Munich, Germany) was added to the culture medium from an aqueous stock solution (10 mM).

### Cloning of α-galactosidase A in an overexpression vector

A plasmid containing the full length cDNA of α-galactosidase A (IRAUp969H0320D, aligned to accession no. NM_000169.2) was obtained from ImaGenes GmbH, Berlin, Germany. Amplification for subcloning was performed using cloned *Pfu* DNA polymerase (Stratagene, La Jolla CA, USA), with the primers 5′-AGGTCGGATCCG ACAATGCAGCTGAGGAACC-3′ (forward) and 5′-GGTGTTCGAATTAAAGTAAGT CTTTTAATGACATCTGCA-3′ (reverse) introducing unique restriction sites for *Bam*HI and *Bst*BI. The amplicon was inserted into mammalian expression vector pcDNA3.1/V5-His_6_ (Invitrogen, Karlsruhe, Germany).

### Site-directed mutagenesis of α-galactosidase A

Expression vectors harbouring α-Gal A mutations were generated by site-directed PCR mutagenesis (3,28) using the QuikChange II XL Site-Directed Mutagenesis Kit (Stratagene, La Jolla, CA, USA). Nucleotide exchanges, deletions or insertions were individually introduced by PCR amplification with *Pfu*Ultra DNA polymerase, the pcDNA3.1/*GLA* plasmid vector containing the wild type sequence was used as template and a 27–37-mer primer set, with sense and antisense primers carrying one of the respective sequence modifications central to their length. Each mutant plasmid was sequenced on a 3130 xl Genetic Analyzer (Applied Biosystems, Darmstadt, Germany).

### Transient expression of mutant enzymes in HEK293H cells

1.5×10^5^ cells were seeded 24 hours before transfection in each well of a 24-well culture plate using 500 µl DMEM medium (Invitrogen, Karlsruhe, Germany) supplemented with 10% Fetal bovine serum (PAA Laboratories, Pasching, Austria). Transient expression of mutant enzymes in HEK293H cells was carried out using Lipofectamine 2000 transfection reagent (Invitrogen, Karlsruhe, Germany), according to the manufacturer's protocol. Typically, prior to transfection, a mixture of plasmid DNA (0.8 µg) and Lipofectamine 2000 transfection reagent (2 µl) in 100 µl of serum-free DMEM or Opti-MEM medium (Invitrogen, Karlsruhe, Germany) was incubated at room temperature for 20 min and applied to the cells thereafter. The cell layer was subsequently incubated for 6 hours at 37°C, the medium containing the transfection reagent was removed and 500 µl fresh DMEM was added. In this step, the DGJ was added where intended. The cells were incubated for another 60 hours and harvested.

### Enzymatic measurement of α-galactosidase A

Cell pellets obtained from confluently grown 24-well cell culture plates were homogenised in 200 µl water and subjected to 5 freeze-thaw cycles using liquid nitrogen. The supernatant collected after centrifugation of the homogenate at 10000× g for 5 min was used in enzyme assays. Protein concentration was measured with the BCA protein assay kit (Thermo Scientific, Braunschweig, Germany) according to the manufacturer's manual. 10 µl of the cell lysates at a concentration of 50 µg/ml were assayed with 20 µl of 4- MU-α-D-galactopyranoside (2 mM, Sigma Aldrich, Munich, Germany) in 0.06 M phosphate citrate buffer (pH 4.7) with some adaptations from the original method described by Desnick et al. [40]. Enzyme reactions were terminated by the addition of 0.2 ml of 1.0 M glycine buffer (pH 10.5), prepared by adjusting the pH using 1.0 M NaOH. The released 4-MU was determined by fluorescence measurement at 360 and 465 nm as the excitation and emission wavelengths respectively, using a microplate fluorescence reader (Tecan, Männedorf, Switzerland). The measured enzyme activity was calculated as nmol 4-MU/mg protein and normalised to one hundred percent wild-type activity.

### Western blot analysis

Western blot analysis for the detection of α-Gal A protein was performed using a custom made rabbit anti-α-Gal A polyclonal antibody from Eurogentec, Cologne, Germany (animals were immunised against peptides QRDSEGRLQADPQRFP (corresponding to amino acids 99–114) and KQGYQLRQGDNFEVWE (corresponding to amino acids 326–341). Furthermore, a mouse GAPDH monoclonal antibody 6C5 (Abcam, Cambridge, UK) was used to visualise GAPDH as an internal loading control.

HEK293H cell lysates were generated by aspirating the media from the 24-well culture plates, washing the cells once with 1× PBS (Biochrom AG, Berlin, Germany) and directly applying 200 µl ice cold RIPA buffer supplemented with protease inhibitor cocktail tablets (Roche Applied Science, Penzberg, Germany) prior to a 20 minute incubation on ice. The cells were then rinsed from the wells, transferred to microcentrifuge tubes and spun at 14000× g for 10 minutes at 4°C to pellet debris. The supernatant was used for the analysis. 50 µg protein were mixed with a suitable volume of 5× Laemmli loading buffer, boiled for 5 minutes on a thermo shaker, centrifuged at 14000× g for 10 minutes at 4°C and loaded on a Criterion precast 4–15% Tris-HCl gel (Bio-Rad, Munich, Germany). Proteins were transferred electrophoretically to a nitrocellulose (Amersham Hybond ECL) membrane (GE Healthcare, Munich, Germany). The membrane was blocked with 5% (w/v) non-fat dried skimmed milk in TBS-Tween 20 [10 mM Tris/HCl (pH 7.5) with 150 mM NaCl and 0.1% Tween 20] at room temperature for 1 hour, and then treated with a primary antibody against GAPDH diluted 1∶10,000 in a milk/blot solution [3% (w/v) non-fat dried skimmed milk in TBS-Tween 20] at 4°C overnight. The blot was then washed three times with excess TBS-Tween 20 and treated with a primary antibody against α-Gal A diluted 1∶500 in the 3% milk/blot solution for 1 hour at room temperature. After another wash procedure, a secondary antibody mix of an Alexa Fluor labelled 680 goat anti-rabbit IgG antibody (Molecular Probes, Karlsruhe, Germany) and an IRDye800 conjugated goat anti-mouse IgG antibody (Rockland-Biomol, Hamburg, Germany) both diluted 1∶10,000 in the 3% milk/blot solution was applied to the membrane. Following extensive washing with TBS-Tween 20, protein bands were visualised by an Odyssey Infrared Imager (Li-Cor Biosciences, Linocln, NE, USA). Quantification and protein size determination were performed using the Odyssey software.

### Determination of kinetic properties of α-galactosidase A mutants

α-Gal A mutants were overexpressed in HEK293H cells. 60 hours after transfection in 24-well plates, the cells were harvested in 100 µl H_2_O and the contents of two wells were pooled. Cells were lysed by 5 freeze-thaw cycles and subsequent centrifugation to obtain a cell-debris free lysate. Quantitative Western blot analysis was carried out to reveal the ratio of the mutant enzyme : wild type. The amount of lysate required to obtain equal amounts of enzyme was calculated. Respective quantities of wild type and mutant enzyme were subjected to the kinetic evaluation described earlier [41]. Substrate concentrations of 0.5, 1, 2, 4, 8 and 28 mM were assayed with constant amount of enzyme in a Lineweaver Burk plot in order to obtain kinetic parameters. To prove that equal amounts of input enzyme (mutants and wild type) were employed for the assay, α-Gal A content of the lysates was controlled with another Western blot before applying the substrate. (Figure S2A). For standard curve, 5, 10, 20, 50 and 100 ng of agalsidase alpha (Replagal, Shire Human Genetic Therapeutics, Dublin, Ireland) were subjected to Western blot analysis and fluorescence values were plotted (see Figure S2B). In the individual experiments between 2 and 6 ng of enzyme were employed.

### Lyso-Gb3 determination

As reference standards (Matreya LLC, USA; purity >98%) we used lyso-ceramide trihexoside ( = lyso-globotriaosylsphingosine = lyso-Gb3). Internal standards were D5-fluticasone propionate (EJY Tech., Inc. Rockville, MD, USA).

We used a gradient HPLC method on a reversed phase column (ACE 3 C8, 50×2.1 mm). The two HPLC pumps and the column oven PE Series 200 were provided by Perkin Elmer, USA. The mass spectrometer used was an API 4000 Q-Trap supplied by Applied Biosystems, USA. The following experimental conditions were used: column temperature 60°C, flow at 0.9 mL/min, injection volume 10 µL, mobile phase with 50 mM formic acid in water (A) and 50 mM formic acid in acetonitrile/acetone (1/1 = v/v; B), gradient at 5% B from 0 to 0.3 minutes, followed by a linear gradient up to 73% B (0.3 to 2.6 minutes) and further on to 100% B (2.6 to 5.7 minutes). From 5.7 to 6.7 minutes 100% B was used. Re-equilibration was done from 6.7 to 7.5 minutes at 5% B. ESI in positive mode was used for peak detection.

The detection mode was MRM, the vaporizer temperature was set at 500°C, ionisation voltage was 5.5 kV, curtain gas pressure was 40 psi. Lyso-Gb3 quantifier was 786.6 to 282.2 m/z and 506.3 to 313.0 m/z for the internal standard D5-fluticasone propionate.

For the sample analysis, 50 µl aliquots were used. 100 µl of internal standard working solution (in ethanol) were added. Samples were mixed for about 30 seconds and centrifuged at 4,000 rpm for 2 minutes. The clear supernatant was transferred into appropriate auto sampler vials which were closed thereafter with crimp caps. Normal and pathological values were determined in 145 healthy controls and 275 Fabry patients with genetically confirmed disease.

### Selection of mutations

The following criteria influenced the decisions whether a given mutant was to be included in the study:

1. The mutation was novel or has not been well described (no biochemical activity data were available; N = 138)
2. The mutation lead to a single amino acid substitution (N = 147)
3. Patient-derived data (phenotype severity, biomarker lyso-Gb3) are available (N = 100)
4. Three sites (R118, S126, D264) were selected for “permuting” the native amino acid into every possible residue resulting from single nucleotide exchanges. R118C and S126G are involved in variant forms of the FD phenotype and found to be more prevalent (9, own data). D264V/Y are involved in classical FD (N = 15)

### Statistical analysis

We used Sperman's rho (r_s_) rank correlation coefficient to test associations between GLA level and enzyme activity (Figure 1). Linear trend tests were used to test associations between two ordinal variables, for example enzyme activity class and DGJ responsiveness (Table 1). To compare the predictive values of female lyso-Gb3 values, male lyso-Gb3 values, *in vitro* enzyme activity and PolyPhen2-scores with regard to clinical phenotype on the mutation level we used ordinal regression with only one of the four different prediction measures as the independent variable and with ‘clinical phenotype’ as the outcome. This regression analysis was performed for all mutations where values on specific prediction measure and the outcome were available. For comparison we repeated the regression analysis for the 21 mutations of which values for all four measures were available (Table 2). We used Nagelkerke's R-Square as a measure of explained variance, the −2 Log-Likelihood as a measure of Goodness of Fit of the model and the proportion of correct classified mutations. All analyses were done using IBM SPSS Statistics, Release 20.0.0 (SPSS, Inc., 2011, Chicago, IL, www.spss.com).

## Supporting Information

Figure S1 Detailed view of the employed analysis. Applied to analysis were residual enzyme activity with (black bars) or without (grey bars) the addition of 20 µM DGJ to the medium and the respective change in cellular protein level semi-quantitatively calculated on a fluorescence reader. The Western Blot method used here was not able to detect endogenously expressed α-Gal A enzyme. Values are mean ± SEM. *p<0.05, **p<0.01, ***p<0.005.
(TIF)

Figure S2 **A**. Control Western Blot. Semiquantitative analysis of α-Gal A level determined by Western Blot were used to examine kinetic properties of the mutant enzymes compared to the wild type. **B**. Recombinant enzyme agalsidase alpha used for Enzyme Replacement Therapy was kindly provided by Shire Human Genetics Therapies to estimate total amount subjected to the kinetic assay [see Material and Methods section]
(TIF)

Table S1 Overview of mutations tested *in vitro* for enzyme activity. Number of experiments (enzyme activity) and patient numbers (lyso-Gb3) are indicated in brackets. The α-Gal A activity limit of quantification (LOQ) in HEK293H cells was defined as 235.3 nmol 4-MU/mg protein for untreated mutations and 292.5 nmol 4-MU/mg protein for 20 µM DGJ treated mutations, respectively, which accounts for 95% of the values obtained from empty vector only transfections. Number of experiments (enzyme activity) and patient numbers (lyso-Gb3) are indicated in brackets. Note that even though females have much lower values of lyso-Gb3 all 6 mutations that caused no elevated lyso-Gb3 in males likewise caused no elevation in females (where applicable), indicating that these mutations may not lead to an accumulation. ^φ^Disease phenotype is conventionally divided into classic, variant and classic/variant. The latter is used for mutations where variant and classic types of FD are reported or a mild classical phenotype is observed. ^Ψ^Mutations have not been described in patients yet. **F396Y was terminated from HGMD. Not a genomic mutation is responsible for the finding. As underlying mechanism RNA editing was proposed.
(DOC)

Table S2 Association of *in vitro* enzyme activity with biochemical and crystallographic data. *In vitro* enzyme activity is associated with the responsiveness to pharmacological chaperone DGJ. As another biochemical parameter DGJ responsiveness is demonstrated to be associated to residual enzyme activity. Enzyme activity shows only a weak linear trend with the parameter “accessible surface area” obtained from crystallographic studies. Accessible surface area is defined as the “average accessibility of each atom in the residue” [33]. However, this model does not take active site residues with a usually high surface accessibility that display low residual activity into consideration. Cut points for accessible surface area were extracted from Garman (2007) [33].
(DOC)

Table S3 Lyso-Gb3 values of classic or presumed classic mutations. Lyso-Gb3 was measured in male and female Fabry patients. The mean is displayed in the table. Generally males have much higher lyso-Gb3-levels than females. With three exceptions (data obtained from one female patient harbouring the mutation *p.A20P*, p.W262*, p.W399*, respectively) all of the mutations shown here caused elevated lyso-Gb3 values above the pathological cut-off of 0.9 ng/ml. * All females are heterozygotes.
(DOC)

Table S4 Kinetic properties of α-Gal A mutants. Asterisks indicate a significant change towards the wild type enzyme (p<0.05). Agalsidase alfa has been tested to validate comparability of the assay.
(DOC)

Table S5 Putative impact on splicing. **A**: Effect of missense mutations on natural acceptor and donor splicing sites. Analysis of the splice-sites revealed 3 possible splice-site abolishments. **B**: Effect of missense mutations on cryptic and novel acceptor and donor sites. The analysis suggested possible changes in 3 sites involving the activation of cryptic and novel donor/acceptor sites. Note: the range of values is given in parenthesis for each splicing algorithm.
(DOC)

Text S1 Additional method. Description of the method applied to reveal alterations of mRNA splicing.
(DOC)
