# Supplementary figures and images for: Functional Characterisation of Alpha-Galactosidase A Mutations as a Basis for a New Classification System in Fabry Disease
Source: PLoS Genet. 2013 Aug 1;9(8):e1003632. doi: 10.1371/journal.pgen.1003632 (PMC3731228; doi:10.1371/journal.pgen.1003632)

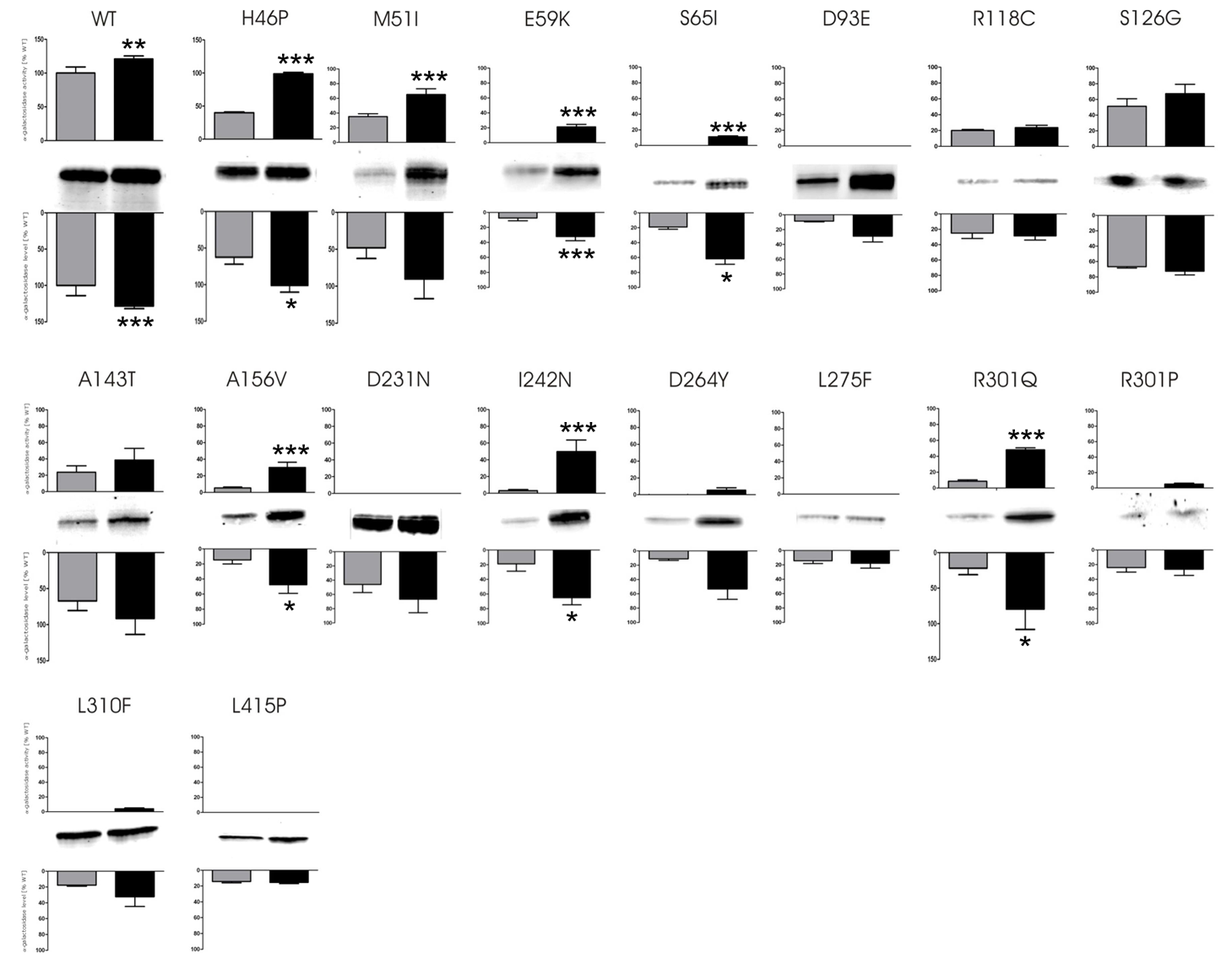

Supplement: Figure S1 — Detailed view of the employed analysis. Applied to analysis were residual enzyme activity with (black bars) or without (grey bars) the addition of 20 µM DGJ to the medium and the respective change in cellular protein level semi-quantitatively calculated on a fluorescence reader. The Western Blot method used here was not able to detect endogenously expressed α-Gal A enzyme. Values are mean ± SEM. *p<0.05, **p<0.01, ***p<0.005. (TIF) [file pgen.1003632.s001.tif]

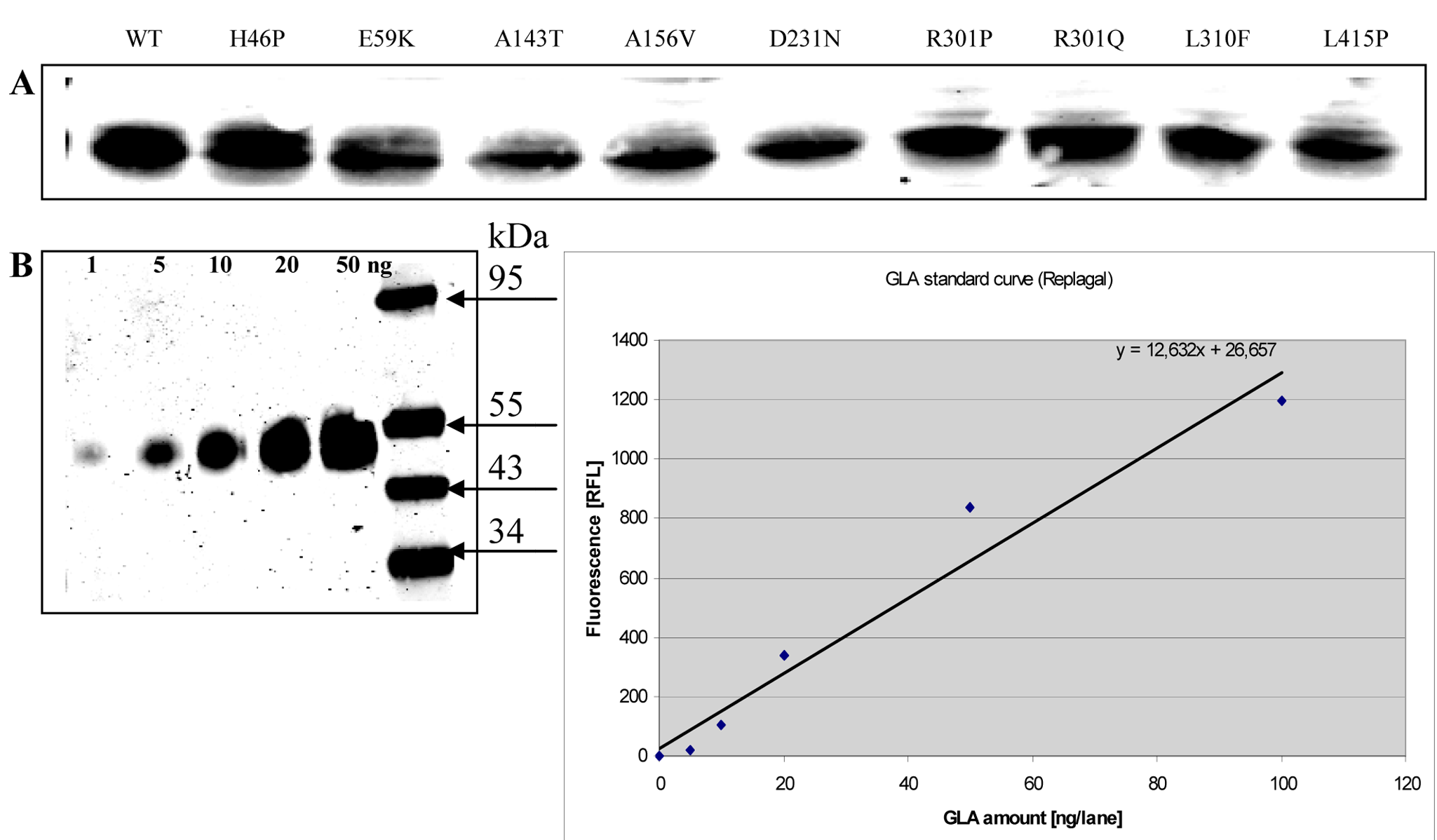

Supplement: Figure S2 — A. Control Western Blot. Semiquantitative analysis of α-Gal A level determined by Western Blot were used to examine kinetic properties of the mutant enzymes compared to the wild type. B. Recombinant enzyme agalsidase alpha used for Enzyme Replacement Therapy was kindly provided by Shire Human Genetics Therapies to estimate total amount subjected to the kinetic assay [see Material and Methods section] (TIF) [file pgen.1003632.s002.tif]
