# Supplementary material for: Functional Characterisation of Alpha-Galactosidase A Mutations as a Basis for a New Classification System in Fabry Disease
Source: PLoS Genet. 2013 Aug 1;9(8):e1003632. doi: 10.1371/journal.pgen.1003632 (PMC3731228; doi:10.1371/journal.pgen.1003632)
Supplement: Table S1 — Overview of mutations tested in vitro for enzyme activity. Number of experiments (enzyme activity) and patient numbers (lyso-Gb3) are indicated in brackets. The α-Gal A activity limit of quantification (LOQ) in HEK293H cells was defined as 235.3 nmol 4-MU/mg protein for untreated mutations and 292.5 nmol 4-MU/mg protein for 20 µM DGJ treated mutations, respectively, which accounts for 95% of the values obtained from empty vector only transfections. Number of experiments (enzyme activity) and patient numbers (lyso-Gb3) are indicated in brackets. Note that even though females have much lower values of lyso-Gb3 all 6 mutations that caused no elevated lyso-Gb3 in males likewise caused no elevation in females (where applicable), indicating that these mutations may not lead to an accumulation. φDisease phenotype is conventionally divided into classic, variant and classic/variant. The latter is used for mutations where variant and classic types of FD are reported or a mild classical phenotype is observed. ΨMutations have not been described in patients yet. **F396Y was terminated from HGMD. Not a genomic mutation is responsible for the finding. As underlying mechanism RNA editing was proposed. (DOC) [file pgen.1003632.s003.doc]

**Supplementary Table S**1:

| **amino acid** | **cDNA** | ***in vitro* enzyme activity [% WT] in mean ± SEM (N)** | | **Ratio**  **DGJ/untreated** | **Lyso-Gb3 [mean (N)]** | | **clinical phenotype**^φ^ | PolyPhen2 | Reference |
| --- | --- | --- | --- | --- | --- | --- | --- | --- | --- |
|  |  | **-DGJ +DGJ** | |  | **male female** | |  |  |  |
| *p.E7** | c19G>T | 0(3) | 0(3) | n.d. |  |  |  |  | Dobrovolny (2008) Am Soc Hum Genet Meet Abs . 150 |
| *p.A15E* | c44C>A | 0(3) | 0(3) | n.d. | 40.7 (2) |  |  | benign | own data |
| *p.W24C* | c72G>T | 45.6±11.2(3) | 89.8±21.0(3) | 2.0 |  |  |  | benign | own data |
| *p.G35R* | c103G>C | 17.9±2.1(3) | 72.3±4.7(3) | 4.0 | 68.9 (3) | 6.8 (4) | variant | probably damaging | Davies (1994) Hum Mol Genet 3:667 |
| *p.L36S* | c107T>C | 0(3) | 0(3) | n.d. |  |  |  | probably damaging | Erdos (2008) Mol Genet Metab 95:224 |
| *p.A37T* | c109G>A | 69.6±13.6(3) | 132.9±27.8(3) | 1.9 | 1.6 (1) |  |  | probably damaging | own data |
| *p.M42V* | c124A>G | 0(3) | 11.9±2.0(3) | n.d. | 111.0 (1) |  | classic | probably damaging | Davies (1996) Eur J Hum Genet 4:219 |
| *p.G43S* | c127G>A | 0(3) | 0(4) | n.d. |  |  |  | probably damaging | own data |
| *p.H46P* | c137A>C | 40.1±1.2(3) | 98.8±2.1(3) | 2.5 |  |  |  | benign | Hwu (2009) Hum Mutat 30:1397 |
| *p.R49G* | c145C>G | 0(3) | 0(3) | n.d. | 75.8 (1) | 7.3 (3) | classic | probably damaging | Germain (2002) Mol Med 8:306 |
| *p.R49C* | c145C>T | 0(3) | 5.1±0.5(3) | n.d. | 89.9 (1) | 21.5 (1) |  | probably damaging | own data |
| *p.M51K* | c152T>A | 0(4) | 8.7±4.4(4) | n.d. |  |  | classic | benign | Ashley (2001) J Hum Genet 46:192 |
| *p.M51I* | c153G>A | 37.4±5.9(7) | 62.0±16.2(3) | 1.7 |  |  | variant | benign | Spada (2006) Am J Hum Genet 79:31 |
| *p.C52W* | c156C>G | 0(3) | 0(3) | n.d. |  |  |  | probably damaging | own data |
| *p.E59K* | c175G>A | 2.2±0.5(8) | 18.5±2.9(4) | 14.2 |  |  | classic | benign | Eng (1994) Hum Mol Genet 3:1795 |
| *p.C63Y* | c188G>A | 0(3) | 0(3) | n.d. | 77.3 (1) | 5.4 (2) |  | probably damaging | Schafer (2005) Hum Mutat 25:412 |
| *p.S65I* | c194G>T | 0(4) | 11.2±1.2(4) | n.d. |  |  |  | probably damaging | own data |
| *p.E66K* | c196G>A | 6.8±0.3(3) | 18.3±2.3(3) | 2.7 | 6.1 (2) |  | classic | probably damaging | Schafer (2005) Hum Mutat 25:412 |
| *p.L68F* | c202C>T | 0(3) | 4.5±1.8(3) | n.d. | 68.4 (1 ) |  | classic | probably damaging | Shabbeer (2002) Mol Genet Metab 76:23 |
| *p.A73V* | c218C>T | 44.0±8.0(8) | 64.7±8.3(3) | 1.5 |  |  | variant | probably damaging | Spada (2006) Am J Hum Genet 79:31 |
| *p.K82** | c244A>T | 0(3) | 0(4) | n.d. |  |  |  |  | Dobrovolny (2008) Am Soc Hum Genet Meet Abs . 150 |
| *p.D83N* | 247G>A | 62.9±12.8(4) | 71.6±9.7(4) | 1.1 |  | 0.5 (1) | variant | probably damaging | own data |
| *p.Y86D* | c256T>G | 0(3) | 0(4) | n.d. |  |  |  | probably damaging | Dobrovolny (2008) Am Soc Hum Genet Meet Abs . 150 |
| *p.L89H* | c266T>A | 0(3) | 0(3) | n.d. |  |  |  | possibly damaging | own data |
|  | c276_277insTGAT | 0(3) | 0(4) | n.d. |  |  |  |  | own data |
| *p.D93Y* | c277G>T | 0(3) | 0(3) | n.d. |  |  |  | probably damaging | own data |
| *p.D93E* | c279C>G | 0(3) | 0(3) | n.d. |  |  |  | possibly damaging | own data |
| *p.W95L* | c284G>T | 0(3) | 0(3) | n.d. |  |  |  | probably damaging | Dobrovolny (2008) Am Soc Hum Genet Meet Abs . 150 |
| *p.W95C* | c285G>T | 0(3) | 0(3) | n.d. |  |  |  | probably damaging | own data |
| *p.R100T* | c299G>C | 0(4) | 0(4) | n.d. |  |  |  | probably damaging | Eng (1997) Mol Med 3:174 |
| *p.S102L* | c305C>T | 71.6±4.4(4) | 78.9±8.3(4) | 1.1 |  | 0,5 (1) |  | benign | own data |
| *p.E103** | c307G>T | 0(3) | 0(4) | n.d. |  |  |  |  | Dobrovolny (2008) Am Soc Hum Genet Meet Abs . 150 |
| *p.R112C* | c334C>T | 0(5) | 0(3) | n.d. | 20.2 (1) | 3,6 (5) | classic | probably damaging | Ishii (1992) Hum Genet 89:29 |
| *p.R112H* | c335G>A | 1.6±0.6(3) | 19.4±2.3(3) | n.d. | 2.2 (2) | 1.0 (1) | variant | probably damaging | Eng (1994) Hum Mol Genet 3:1795 |
| *p.R118S* | c352C>A | 76.2±9.5(3) | 70.9±2.6(3) | n.d. |  |  |  | benign | Ψ |
| *p.R118G* | c352C>G | 37.6±4.1(3) | 44.0±5.0(3) | 1.2 |  |  |  | benign | Ψ |
| *p.R118C* | 352C>T | 20.0±1.3(3) | 23.7±2.9(4) | 1.2 | 0.6 (1) | 0.5 (1) | variant | possibly damaging | Spada (2006) Am J Hum Genet 79:31 |
| *p.R118H* | c353G>A | 67.4±3.4(3) | 66.9±3.1(3) | n.d. |  |  |  | benign | Ψ |
| *p.R118P* | c353G>C | 49.0±4.9(3) | 47.7±2.0(3) | n.d. |  |  |  | benign | Ψ |
| *p.R118L* | c353G>T | 51.7±3.8(3) | 51.4±4.0(3) | n.d. |  |  |  | benign | Ψ |
| *p.L120V* | c358C>G | 50.1±5.0(3) | 62.0±2.6(3) | 1.2 |  |  |  | probably damaging | Chien (2009) Hum Genet 125:336 [2] |
| *p.H125P* | c374A>C | 0(3) | 0(3) | n.d. |  |  |  | probably damaging | Auray-Blais (2008) Mol Genet Metab 93:331 |
| *p.S126R* | c376A>C | 19.7±1.2(6) | 23.5±2.3(4) | 1.2 |  |  |  | possibly damaging | Ψ |
| *p.S126G* | c376A>G | 51.3±9.6(4) | 67.4±11.9(4) | 1.3 | 0.5 (4) | 0.5 (8) | variant | benign | Branton (2002) Medicine (Baltimore) 81:122 |
| *p.S126C* | c376A>T | 2.1± 0.4(3) | 2.7±0.2(3) | n.d. |  |  |  | possibly damaging | Ψ |
| *p.S126N* | c377G>A | 14.2±2.4(3) | 17.0±2.3(3) | 1.2 |  |  |  | benign | Ψ |
| *p.S126T* | c377G>C | 48.9±11.4(3) | 42.8±7.9(4) | n.d. |  |  |  | benign | Ψ |
| *p.S126I* | c377G>T | 40.5±8.1(4) | 43.2±7.7(4) | 1.1 |  |  |  | possibly damaging | Ψ |
| *p.L129P* | c386T>C | 0(3) | 0(3) | n.d. | 72.1 (2) | 7.9 (2) | classic/variant | probably damaging | Whybra (2001) J Inherit Metab Dis 24:715 |
| *p.L131P* | c392T>C | 0(3) | 0(3) | n.d. | 68.6 (4) | 7.0 (2) | classic | probably damaging | Eng (1994) Hum Mol Genet 3:1795 |
| *p.G132R* | c394G>A | 0(3) | 0(3) | n.d. |  |  | classic | probably damaging | Shabbeer (2002) Mol Genet Metab 76:23 |
| *p.G132E* | c395G>A | 0(3) | 0(3) | n.d. | 119.0 (1) | 12.3 (1) |  | probably damaging | Kwan (2007) Am Soc Hum Genet Meet Abs .:1537 |
| *p.A135V* | c404C>T | 0(3) | 6.9±2.4(3) | n.d. | 104.2 (2) |  | classic | benign | Shabbeer (2005) Hum Mutat 25:299 |
| *p.D136E* | c408T>A | 0(3) | 31.3±3.2(3) | n.d. | 42.3 (1) | 6.6 (2) |  | probably damaging | own data |
| *p.G138R* | c412G>A | 0(3) | 0(3) | n.d. |  | 3.6 (1) | classic | probably damaging | Eng (1997) Mol Med 3:174 |
| *p.N139S* | c416A>G | 147.8±28.3(3) | 176.4±36.4(3) | 1.2 | 88.6 (1) | 3.0 (4) | variant | benign | Havndrup (2010) Eur J Heart Fail 12:535 |
| *p.T141I* | c422C>T | 0(3) | 0(3) | n.d. |  |  | classic | probably damaging | Shabbeer (2002) Mol Genet Metab 76:23 |
| *p.C142R* | c424T>C | 0(3) | 0(3) | n.d. |  |  | classic | probably damaging | Topaloglu (1999) Mol Med 5:806 |
| *p.A143T* | c427G>A | 31.3±5.6(7) | 49.4±4.8(6) | 1.6 | 0.5 (8) | 0.5 (12) | classic/variant | benign | Eng (1997) Mol Med 3:174 |
| *p.A143P* | c427G>C | 0(3) | 0(5) | n.d. |  |  | classic | possibly damaging | Eng (1994) Hum Mol Genet 3:1795 |
| *p.G147R* | c439G>A | 0(3) | 0(3) | n.d. |  | 16.2 (1) |  | probably damaging | Schafer (2005) Hum Mutat 25:412 |
| *p.A156D* | c467C>A | 0(3) | 0(3) | n.d. |  |  |  | probably damaging | own data |
| *p.A156V* | c467C>T | 4.3±1.0(9) | 16.8±2.3(5) | 3.9 |  |  | classic | probably damaging | Okumiya (1995) Hum Genet 95:557 |
| *p.W162G* | c484T>G | 0(3) | 5.2±1.2(3) | n.d. | 42.5 (3) | 6.8 (3) |  | probably damaging | own data |
| *p.D165H* | c493G>C | 3.4±1.1(3) | 11.9±1.1(3) | 3.5 |  | 3.7 (1) |  | probably damaging | Rakoczi (2007) Orv Hetil 148:1087 |
| *p.D165Y* | c493G>T | 0(3) | 0(3) | n.d. |  |  |  | probably damaging | own data |
| *p.D165V* | c494A>T | 0(4) | 0(4) | n.d. | 9.1 (1) |  | classic | probably damaging | Davies (1994) Hum Mol Genet 3:667 |
| *p.D170N* | c508G>A | 0(3) | 0(3) | n.d. | 72.1 (1) | 4.8 (1) |  | probably damaging | own data |
| *p.C172G* | c514T>G | 0(3) | 4.4±2.0(3) | n.d. | 59.3 (1) | 21.7 (1) | classic | probably damaging | Yasuda (2003) Hum Mutat 22:486 |
| *p.C172Y* | c515G>A | 0(3) | 0(3) | n.d. | 80.2 (1) | 5.6 (1) | classic | probably damaging | Eng (1994) Hum Mol Genet 3:1795 |
|  | c521insT | 0(3) | 0(3) | n.d. |  | 5.9 (2) |  |  | own data |
| *p.D175N* | c523G>A | 70.0±2.1(3) | 84.3±8.5(3) | 1.2 |  |  |  | benign | own data |
| *p.D175E* | c525C>G | 89.8±2.2(3) | 89.0±7.3(3) | n.d. |  | 0.5 (1) |  | benign | own data |
|  | c525_526delCA | 0(3) | 0(3) | n.d. |  |  |  |  | own data |
| *p.G183V* | c548G>T | 0(3) | 6.7±2.1(4) | n.d. |  |  |  | probably damaging | Dobrovolny (2008) Am Soc Hum Genet Meet Abs . 150 |
| *p.M187T* | c561G>C | 0(3) | 0(4) | n.d. |  | 2.9 (1) | classic | possibly damaging | Shabbeer (2006) Hum Genomics 2:297 |
| *p.L191P* | c572T>C | 0(3) | 0(3) | n.d. |  | 4.9 (1) | classic | probably damaging | Cooper (2000) Hum Genet 107:535 |
| *p.W204C* | c612G>C | 0(3) | 4.4±0.9(3) | n.d. |  | 8.9 (1) |  | probably damaging | own data |
| *p.K213M* | c638A>T | 83.4±29.6(5) | 82.5±15.5(5) | n.d. | 9.3 (1) |  |  | possibly damaging | own data |
| *p.N215S* | c644A>G | 39.5±1.5(6) | 63.9±3.3(6) | 1.6 | 4.2 (7) | 1.1 (6) | variant | benign | Davies (1993) Hum Mol Genet 2:1051 |
| *p.I219T* | c656T>C | 53.3±2.2(3) | 85.3±10.0(3) | 1.6 |  |  |  | benign | Hwu (2009) Hum Mutat 30:1397 |
| *p.R220Q* | c659G>A | 104.5±11.3(4) | 144.0±11.9(4) | 1.4 |  |  |  | benign | own data |
| *p.N224S* | c671A>G | 31.1±2.4(3) | 82.2±2.2(3) | 2.6 |  | 5.3 (3) | classic | probably damaging | Ashton-Prolla (2000) J Investig Med 48:227 |
| *p.H225D* | c673C>G | 32.2±8.1(3) | 60.5±19.1(3) | 1.9 | 2.8 (1) |  |  | possibly damaging | own data |
| *p.H225R* | c674A>G | 0(7) | 3.0±0.8(4) | n.d. |  |  | classic | possibly damaging | Politei (2005) Rev Neurol 41:506 |
| *p.R227Q* | c680G>A | 0(3) | 0(3) | n.d. | 53.7 (5) | 11.2 (3) | classic | probably damaging | Eng (1993) Am J Hum Genet 53:1186 |
| *p.D231N* | c691G>A | 0(3) | 0(3) | n.d |  | 5.9 (1) | classic | probably damaging | Redonnet-Vernhet (1996) J Med Genet 33:682 |
| *p.I232T* | c695T>C | 11.5±2.8(3) | 61.6±7.2(5) | 5.1 |  |  |  | possibly damaging | own data |
| *p.W236C* | c708G>C | 0(3) | 0(3) | n.d. | 95.8 (1) | 7.8 (2) | classic | probably damaging | Davies (1996) Eur J Hum Genet 4:219 |
| *p.S238N* | c713G>A | 36.0±6.7(4) | 94.3±24.5(4) | 2.6 |  |  | variant | probably damaging | Monserrat (2007) J Am Coll Cardiol 50:2399 |
| *p.I242N* | c725T>A | 3.1±1.4(4) | 49.8±13.9(4) | 16.1 |  |  | classic | probably damaging | Takata (1997) Brain Dev 19:111 |
| *p.R252T* | c755G>C | 117.0±10.0(4) | 134.3±22.5(4) | 1.1 |  | 0.5 (4) |  | benign | own data |
| *p.I253S* | c758T>G | 4.4±1.6(5) | 53.4±7.1(5) | 10.3 |  |  |  | probably damaging | own data |
| *p.G261V* | c782G>T | 0.2±1.4(5) | 3.5±1.1(5) | n.d. | 84.4 (2) |  |  | probably damaging | own data |
| *p.W262C* | c786G>C | 0(3) | 0(3) | n.d. | 9.4 (1) | 7.7 (4) | classic/variant | probably damaging | Schafer (2005) Hum Mutat 25:412 |
| *p.D264N* | c790G>A | 37.9±3.7(3) | 84.5±6.4(3) | 2.2 |  |  |  | probably damaging | Ψ |
| *p.D264H* | c790G>C | 1.7±0.9(3) | 10.2±2.9(3) | 6.0 |  |  |  | probably damaging | Ψ |
| *p.D264Y* | c790G>T | 0(3) | 5.4±2.9(3) | n.d. | 98.9 (1) | 13.7 (3) | classic | probably damaging | Shabbeer (2005) Hum Mutat 25:299 |
| *p.D264A* | c791A>C | 0(3) | 0(3) | n.d. |  |  |  | probably damaging | Ψ |
| *p.D264G* | c791A>G | 0(3) | 0(3) | n.d. |  |  |  | probably damaging | Ψ |
| *p.D264V* | c791A>T | 0(3) | 0(3) | n.d. | 59.3 (1) |  | classic | probably damaging | Eng (1993) Am J Hum Genet 53:1186 |
| *p.D264E* | c792C>A | 0(3) | 0(3) | n.d. |  |  |  | probably damaging | Ψ |
| *p.M267T* | c800T>C | 27.5±4.4(4) | 30.5±1.8(4) | 1.2 | 2.7 (1) |  |  | probably damaging | own data |
| *p.L268S* | c803T>C | 0(3) | 10.8±0.7(3) | n.d. | 18.0 (1) | 3.0 (1) | classic | probably damaging | Schafer (2005) Hum Mutat 25:412 |
| *p.V269M* | c805G>A | 0(4) | 17.3±1.4(4) | n.d. |  |  | classic | probably damaging | Shabbeer (2006) Hum Genomics 2:297 |
| *p.V269A* | c806T>C | 9.0±1.4(9) | 45.0±4.3(4) | 5.0 |  |  | classic | possibly damaging | Davies (1993) Hum Mol Genet 2:1051 |
|  | c807delG | 0(3) | 0(3) | n.d. |  |  |  |  | own data |
| *p.I270M* | c810T>G | 2.3±1.7(4) | 33.7±16.8(4) | 14.7 |  |  |  | probably damaging | own data |
| *p.N272S* | c815A>G | 0(3) | 0(3) | n.d. |  |  | classic | probably damaging | Verovnik (2004) Eur J Hum Genet 12:678 |
| *p.F273L* | c819T>A | 0(6) | 0(3) | n.d. |  |  | variant | probably damaging | Zhang. SH et al.. 2007 |
| *p.L275F* | c823C>T | 0(4) | 0(3) | n.d. |  | 3.9 (1) | classic/variant | probably damaging | Dobrovolny (2008) Am Soc Hum Genet Meet Abs . 150 |
| *p.L275H* | c824T>A | 0(5) | 0(5) | n.d. |  |  |  | probably damaging | own data |
| *p.T282I* | c845C>T | 5.0±0.5(3) | 47.7±0.7(3) | 9.5 | 22.6 (2) |  |  | possibly damaging | own data |
| *p.Q283P* | c848A>C | 0(3) | 0(3) | n.d. |  |  | classic | probably damaging | Shabbeer (2006) Hum Genomics 2:297 |
| *p.A285D* | c854C>A | 0(3) | 0(3) | n.d. |  |  | classic | probably damaging | Shabbeer (2006) Hum Genomics 2:297 |
| *p.W287** | c860G>A | 0(3) | 0(3) | n.d. |  |  |  |  | Davies (1993) Hum Mol Genet 2:1051 |
| *p.M290L* | c868A>T | 18.5±8.2(3) | 22.4±9.6(3) | 1.2 |  |  |  | probably damaging | own data |
| *p.M290I* | c870G>A | 39.0±1.8(3) | 70.5±7.2 | 1.8 |  |  | classic | probably damaging | Shabbeer (2006) Hum Genomics 2:297 |
| *p.A291T* | c871G>A | 13.2±3.4(3) | 55.7±0.7(3) | 3.7 |  |  |  | probably damaging | Zhang (2007) J Nephrol 20:716 |
| *p.L294** | c881T>G | 0(4) | 0(3) | n.d. |  |  |  |  | Blaydon (2001) Hum Mutat 18:459 |
| *p.L294S* | c881T>C | 0(3) | 12.4±1.7(3) | n.d. |  | 6.6 (2) |  | probably damaging | own data |
|  | c883delT | 0(3) | 0(3) | n.d. |  |  |  |  | Dobrovolny (2008) Am Soc Hum Genet Meet Abs . 150 |
| *p.S297C* | c890C>G | 0(3) | 3.8±0.7(3) | n.d. | 121.0 (1) |  | classic | probably damaging | Germain (2002) Mol Med 8:306 |
| *p.R301G* | c901C>G | 19.3±4.1(7) | 56.5±3.4(3) | 2.9 |  |  | classic | probably damaging | Lai (2001) Hum Genet 109:469 |
| *p.R301Q* | c902G>A | 8.5±1.8(9) | 48.0±2.6(3) | 5.6 |  | 2.2 (1) | classic/variant | probably damaging | Sakuraba (1990) Am J Hum Genet 47:784 |
| *p.R301P* | c902G>C | 0(4) | 5.0±1.3(3) | n.d. |  | 10.7 (1) | classic | probably damaging | Ashley (2001) J Hum Genet 46:192 |
| *p.L310F* | c928C>T | 0(4) | 4.1±1.1(3) | 41.0 |  |  | classic | probably damaging | Calado (2004) Hum Genet 115:347 |
| *p.L311V* | c931C>G | 1.9±0.4(3) | 40.1±4.9(3) | n.d. | 53.6 (1) |  |  | probably damaging | own data |
| *p.D313Y* | c937G>T | 83.9±21.1(4) | 100.3±23.9(3) | 1.2 | 0.5 (27) | 0.5 (36) | variant | possibly damaging | Eng (1993) Am J Hum Genet 53:1186 |
| *p.V316I* | c946G>A | 65.6±22.3(3) | 68.3±21.8(3) | n.d. | 0.7 (1) |  | variant | benign | own data |
| *p.V316G* | c947T>G | 0(3) | 0(3) | n.d. | 103.0 (1) | 13.0 (1) |  | probably damaging | own data |
|  | c948InsT | 0(3) | 0(3) | n.d. | 75.1 (1) | 5.8 (2) |  |  | own data |
| *p.I319T* | c956T>C | 20.2±3.8(3) | 58.3±12.6(3) | 2.9 |  | 3.0 (2) |  | probably damaging | own data |
| *p.N320I* | c959A>T | 2.0±0.5(3) | 31.8±8.9(3) | 17.7 |  | 2.9 (1) | classic | probably damaging | Schafer (2005) Hum Mutat 25:412 |
| *p.Q321H* | c963G>C | 3.3±1.9(3) | 25.3±7.1(3) | 7.7 |  |  |  | probably damaging | own data |
| *p.G325S* | c973G>A | 25.6±9.6(3) | 55.4±14.4(3) | 2.2 |  |  |  | probably damaging | own data |
| *p.Q327K* | c979C>A | 0(4) | 0(5) | n.d. |  |  | classic | probably damaging | Davies (1993) Hum Mol Genet 2:1051 |
| *p.Q327E* | c979C>G | 21.5±2.8(3) | 80.9±10.0(3) | 3.8 |  | 1.7 (1) | classic/variant | probably damaging | Schafer (2005) Hum Mutat 25:412 |
| *p.G328A* | c983G>C | 6.2±1.0(3) | 30.0±6.4(3) | 4.8 |  |  | classic | probably damaging | Eng (1993) Am J Hum Genet 53:1186 |
| *p.E341K* | c1021G>A | 0(3) | 0(3) | n.d. | 64.4 (2) |  | classic | probably damaging | Shabbeer (2002) Mol Genet Metab 76:23 |
| *p.E341D* | c1023A>C | 0(3) | 0(3) | n.d. |  |  | classic | probably damaging | Shabbeer (2002) Mol Genet Metab 76:23 |
| *p.R342Q* | c1025G>A | 0(4) | 0(3) | n.d. |  | 2.6 (3) | classic | possibly damaging | Ploos van Amstel (1994) Hum Mol Genet 3:503 |
| *p.R342L* | c1025G>T | 0(3) | 0(3) | n.d. | 48.3 (2) | 3.0 (2) |  | possibly damaging | own data |
| *p.L344P* | c1031T>C | 0(3) | 0(3) | n.d. | 24.9 (1) |  | classic | probably damaging | Schafer (2005) Hum Mutat 25:412 |
| *p.S345P* | c1033T>C | 0(3) | 13.3±3.8(3) | n.d. | 16.7 (1) |  | classic | benign | Schafer (2005) Hum Mutat 25:412 |
| *p.W349R* | c1045T>A | 1.6±1.4(4) | 8.9±3.9(4) | 5.6 |  |  |  | probably damaging | own data |
| *p.R356W* | c1066C>T | 16.9±2.3(9) | 62.7±3.9(3) | 3.7 |  | 2.3 (2) | classic | possibly damaging | Bernstein (1989) J Clin Invest 83:1390 |
| *p.R356Q* | c1067G>A | 89.1±5.0(3) | 99.4±4.3(3) | 1.1 |  |  |  | benign | Hwu (2009) Hum Mutat 30:1397 |
| *p.G360C* | c1078G>T | 11.9±0.5(3) | 26.5±2.7(3) | 2.2 |  |  |  | probably damaging | Lin (2007) Hum Genet 122:212 |
| *p.G361R* | c1081G>C | 0(3) | 3.7±1.1(3) | n.d. |  |  | classic | probably damaging | Davies (1993) Hum Mol Genet 2:1051 |
| *p.R363H* | c1088G>A | 31.9±2.9(8) | 57.9±5.5(3) | 1.8 |  |  | classic/variant | possibly damaging | Cooper (2000) Hum Genet 107:535 |
| *p.A368T* | c1102G>A | 103.7±33.6(3) | 93.3± 5.0(3) | n.d. |  | 0.5 (3) |  | benign | own data |
| *p.L372P* | c1115T>C | 0(3) | 2,6±0,7(4) | n.d. |  |  |  | probably damaging | own data |
| *p.L372R* | c1115T>G | 0(3) | 0(3) | n.d. |  |  |  | probably damaging | Dobrovolny (2008) Am Soc Hum Genet Meet Abs . 150 |
| *p.G373D* | c1118G>A | 0(4) | 0(4) | n.d. |  |  |  | probably damaging | Germain (2001) Hum Mutat 17:353 |
| *p.C378R* | c1132T>C | 0(3) | 0(3) | n.d. |  |  |  | probably damaging | Shin (2001) ASHG Annual Meeting Abstracts. 1799 |
| *p.I384N* | c1151T>A | 0(3) | 0(3) | n.d. | 25.1(1) | 1.5 (1) | classic | probably damaging | Shabbeer (2002) Mol Genet Metab 76:23 |
| *p.T385A* | c1153A>G | 45.0±3.7(3) | 48.9±7.5(3) | 1.1 |  | 0.5 (1) |  | benign | own data |
| *p.Q386P* | c1157A>C | 0(3) | 0(3) | n.d. |  |  | classic | possibly damaging | Shabbeer (2006) Hum Genomics 2:297 |
| *p.P389L* | C1166C>T | 0(3) | 0(3) | n.d. | 72.9 (1) |  |  | probably damaging | own data |
| *p.V390M* | c1168G>A | 0(3) | 0(4) | n.d. |  |  |  | benign | own data |
| *p.G395A* | c1184G>C | 20.1±1.7(6) | 23.1±2.0(6) | 1.1 |  |  |  | probably damaging | own data |
| *p.F396Y*** | c1187T>A | 87.6±14.2(4) | 93.8±15.7(4) | 1.1 |  |  |  | benign | Novo (1995) Nuceic Acids Research 23. 2636-40 |
| *p.E398A* | c1193A>C | 48.9±4.7(3) | 62.5±7.8(3) | 1.3 |  |  |  | benign | own data |
| *p.S405R* | c1213A>C | 91.0±10.2 (3) | 92.7±11.1 (3) | n.d. |  | 0.5 (1) |  |  | own data |
|  | c1221delA | 0(4) | 0(3) | n.d. |  | 8.2 (2) |  |  | own data |
| *p.T410I* | c1229C>T | 2.3±0.8(4) | 16.1±3.5(4) | n.d. |  |  |  | possibly damaging | own data |
| *p.L415F* | c1243C>T | 83.2±5.9(3) | 99.5±12.2(3) | 1.2 |  | 0.5 (1) |  | benign | own data |
| *p.L415P* | c1244T>C | 0(3) | 0(3) | n.d. |  | 4.7 (1) |  | probably damaging | Serebrinsky (2006) Hum Genet 119:361 |
| *p.Q416X* | c1246C>T | 0(3) | 0(3) | n.d. | 27.7 (3) | 6.7 (1) |  |  | own data |
| *p.Q416P* | c1247A>C | 0(3) | 0(3) | n.d. |  |  |  |  | own data |
| *p.E418G* | c1253A>G | 74.6±15.5(3) | 89.1±4.9(3) | 1.2 | 0.5 (1) |  |  | benign | own data |
