# Supplementary material for: Functional Characterisation of Alpha-Galactosidase A Mutations as a Basis for a New Classification System in Fabry Disease
Source: PLoS Genet. 2013 Aug 1;9(8):e1003632. doi: 10.1371/journal.pgen.1003632 (PMC3731228; doi:10.1371/journal.pgen.1003632)
Supplement: Table S2 — Association of in vitro enzyme activity with biochemical and crystallographic data. In vitro enzyme activity is associated with the responsiveness to pharmacological chaperone DGJ. As another biochemical parameter DGJ responsiveness is demonstrated to be associated to residual enzyme activity. Enzyme activity shows only a weak linear trend with the parameter “accessible surface area” obtained from crystallographic studies. Accessible surface area is defined as the “average accessibility of each atom in the residue” [33]. However, this model does not take active site residues with a usually high surface accessibility that display low residual activity into consideration. Cut points for accessible surface area were extracted from Garman (2007) [33]. (DOC) [file pgen.1003632.s004.doc]

**Supplementary Table S2**:

|  | **enzyme activity** |  |  |  |  |
| --- | --- | --- | --- | --- | --- |
|  | 0% | >0% - 20% | ≥20%-60% | ≥60% | p (for linear trend test) |
| **DGJ responsiveness** |  |  |  |  |  |
| not responsive (n=90) | 69 | 5 | 8 | 8 | <0.001 |
| responsive (n=69) | 14 | 24 | 20 | 11 |  |
| **accessible surface area [A^2^]** |  |  |  |  |  |
| 0-1 (buried, 56) | 38 | 11 | 6 | 1 | 0.121 |
| 1-10 (partially buried, 35) | 20 | 7 | 5 | 3 |  |
| 10-40 (exposed) | 4 | 0 | 1 | 1 |  |
