# Supplementary material for: Functional Characterisation of Alpha-Galactosidase A Mutations as a Basis for a New Classification System in Fabry Disease
Source: PLoS Genet. 2013 Aug 1;9(8):e1003632. doi: 10.1371/journal.pgen.1003632 (PMC3731228; doi:10.1371/journal.pgen.1003632)
Supplement: Table S3 — Lyso-Gb3 values of classic or presumed classic mutations. Lyso-Gb3 was measured in male and female Fabry patients. The mean is displayed in the table. Generally males have much higher lyso-Gb3-levels than females. With three exceptions (data obtained from one female patient harbouring the mutation p.A20P, p.W262*, p.W399*, respectively) all of the mutations shown here caused elevated lyso-Gb3 values above the pathological cut-off of 0.9 ng/ml. * All females are heterozygotes. (DOC) [file pgen.1003632.s005.doc]

**Supplementary Table S3**:

| **amino acid** | **cDNA** | **Lyso Gb3 (mean) Norm < 0.9 ng/ml** | | **mutation class** | **clinical phenotype** | **Reference** |
| --- | --- | --- | --- | --- | --- | --- |
|
|
|
|  |  | **male female*** | |  |  |  |
| *p.Q2** | c4C>T | 13.5 (1) |  | nonsense |  | own data |
|  | c35_58del | 26.9 (1) | 5.3 (3) | small deletion |  | own data |
| *p.A20P* | c58G>C | 8.5 (1) | 0.7 (1) |  | classic | Nakao (1995) N Engl J Med 333:288 |
| *p.W24** | c71G>A |  | 7.4 (2) | nonsense |  | own data |
| *p.L45P* | c134T>C | 42.7 (2) | 9.8 (1) |  |  | own data |
|  | c162delT | 16.9 (1) |  | small deletion |  | Schafer (2005) Hum Mutat 25:412 |
| *p.C56Y* | c167G>A | 33.1 (1) | 3.4 (1) |  |  | Davies (1996) Eur J Hum Genet 4:219 |
| *p.I91T* | c272T>C |  | 0.7 (1) |  |  |  |
| Exon 2 del | g.100545168_100545720del |  | 8.0 (6) | gross deletion |  | Lukas (2012) Clin Kidney J 5: 395–400 |
|  | c354_368del |  | 10.1 (1) | small deletion |  | own data |
|  | c369+1G>A | 65.1 (1) | 5.7 (1) | splice |  | Ashton-Prolla (2000) J Investig Med 48:227 |
| *p.Y151** | c453C>A | 126.0 (1) |  | nonsense |  | Shabbeer (2005) Hum Mutat 25:299 |
| *p.W157** | c469C>T | 16.5 (1) |  | nonsense | classic | Eng (1994) Hum Mol Genet 3:1795 |
| *p.W162C* | c486G>T | 28.5 (1) | 5.6 (1) |  |  | Germain (1996) Hum Genet 98:719 |
| *p.V164G* | c491T>G | 19.5 (1) | 2.1 (1) |  |  | own data |
| *p.L167Q* | c500T>A |  | 10.7 (1) |  |  | own data |
|  | c547+1G>A | 82.7 (3) | 7.3 (3) | splice |  | Ashton-Prolla (2000) J Investig Med 48:227 |
|  | c568delG | 42.9 (2) | 12.9 (1) | small deletion | classic | Shabbeer (2002) Mol Genet Metab 76:23 |
|  | c571delC | 83.2 (1) | 10.6 (1) | small deletion |  | own data |
| *p.C202Y* | c605G>A | 31.8 (1) | 5.5 (1) |  |  | Eng (1997) Mol Med 3:174 |
| *p.W204** | c611G>A | 102.0 (1) | 6.4 (1) | nonsense |  | Ashton-Prolla (2000) J Investig Med 48:227 |
|  | c639+1G>A |  | 5.2 (1) | splice | classic | Rodriguez-Mari (2003) Hum Mutat 22:258 |
| *p.R220X* | c658C>T |  | 3.7 (2) | nonsense | classic | Meaney (1994) Hum Mol Genet 3:1019 |
| *p.R227X* | c679C>T | 50.3 (3) | 6.0 (2) | nonsense | classic | Davies (1993) Hum Mol Genet 2:1051 |
|  | c718_719delAA | 62.6 (1) | 5.4 (1) | small deletion | classic | Davies (1994) Hum Mol Genet 3:667 |
|  | c.719delA | 172.0 (1) |  | small deletion |  | own data |
|  | c742_743delTT | 84.9 (1) | 4.9 (2) | small deletion |  | own data |
|  | c776delC | 30.7 (1) |  | small deletion |  | own data |
|  | c777delA | 45.4 (1) | 13.0 (1) | small deletion |  | Eng (1997) Mol Med 3:174 |
| *p.W262** | c785G>A |  | 0.9 (1) | nonsense |  | Shabbeer (2006) Hum Genomics 2:297 |
|  | c801+2T>C |  | 16.6 (1) | splice | . | Blaydon (2001) Hum Mutat 18:459 |
|  | c802delT | 80.5 (1) | 13.3 (3) | small deletion |  | own data |
| *p.Q280** | c838C>T |  | 6.5 (2) | nonsense |  | own data |
| *p.R301** | c901C>T | 16.4 (2) | 6.3 (3) | nonsense | classic | Eng (1994) Hum Mol Genet 3:1795 |
|  | c.913delC |  | 5.4 (1) | small deletion |  | own data |
| *p.Q312** | c934C>T | 71.7 (2) | 9.3 (2) | nonsense |  | own data |
| *p.Q321** | c961C>T |  | 6.4 (1) | nonsense |  | Schafer (2005) Hum Mutat 25:412 |
|  | c993_994insA | 68.8 (1) | 5.8 (3) | small insertion |  | own data |
| *p.Q333** | c997C>T | 25.7 (1) |  | nonsense |  | Lorenz (2003) Wien Klin Wochenschr 115:235 |
| *p.W340** | c1020G>A |  | 5.5 (1) | nonsense | classic | Eng (1993) Am J Hum Genet 53:1186 |
| *p.R342** | c1025G>A | 82.3 (2) | 4.8 (2) | nonsense | classic | Ploos van Amstel (1994) Hum Mol Genet 3:503 |
| *p.R342P* | c1025G>C |  | 3.3 (1) |  |  | own data |
|  | c1029-1030delTC | 94.2 (1) |  | small deletion |  | own data |
| *p.S345** | c1034C>A | 119.0 (1) |  | nonsense |  | own data |
| *p.W349** | c1046G>A | 66.2 (1) |  | nonsense |  | Ashley (2001) J Hum Genet 46:192 |
| *p.Q357** | c1069C>T | 122.0 (1) |  | nonsense |  | Cooper (2000) Hum Genet 107:535 |
| *p.E358** | c1072G>T |  | 7.6 (1) | nonsense |  | own data |
| *p.Y397** | c1191T>G |  | 9.4 (1) | nonsense |  | own data |
| *p.W399** | c1196G>A | 88.5 (3) | 0.5 (1) | nonsense | classic | Eng (1997) Mol Med 3:174 |
|  | c1208delT | 66.2 (1) | 6.1 (2) | small deletion | classic | Germain (2002) Mol Med 8:306 |
|  | c1235-1236delCT | 21.3 (4) | 5.2 (4) | small deletion |  | Blaydon (2001) Hum Mutat 18:459 |
|  | c1277-1278delAA |  | 6.5 (1) | small deletion |  | Yasuda (2003) Hum Mutat 22:486 |
