# Supplementary material for: Functional Characterisation of Alpha-Galactosidase A Mutations as a Basis for a New Classification System in Fabry Disease
Source: PLoS Genet. 2013 Aug 1;9(8):e1003632. doi: 10.1371/journal.pgen.1003632 (PMC3731228; doi:10.1371/journal.pgen.1003632)
Supplement: Table S4 — Kinetic properties of α-Gal A mutants. Asterisks indicate a significant change towards the wild type enzyme (p<0.05). Agalsidase alfa has been tested to validate comparability of the assay. (DOC) [file pgen.1003632.s006.doc]

**Supplementary Table S4**:

| Mutation | K_M_ (mM) | v_max_ (mmol MU/mg protein/h) |
| --- | --- | --- |
| Agalsidase alfa | 3.7 | 1.8 |
| Wild-type | 2.9 | 2.0 |
| H46P | 8.6* | 2.8 |
| R49G | n.d. | n.d. |
| E59K | 5.0* | 1.6 |
| S65I | n.d. | n.d. |
| A143T | 3.6 | 3.7 |
| A156V | 1.8 | 1.1 |
| D231N | n.d. | n.d. |
| R301P | 2.4 | 0.3* |
| R301Q | 3.5 | 1.7 |
| L310F | 4.6 | 1.0 |
| L415P | n.d. | n.d. |
