# Supplementary material for: Functional Characterisation of Alpha-Galactosidase A Mutations as a Basis for a New Classification System in Fabry Disease
Source: PLoS Genet. 2013 Aug 1;9(8):e1003632. doi: 10.1371/journal.pgen.1003632 (PMC3731228; doi:10.1371/journal.pgen.1003632)
Supplement: Table S5 — Putative impact on splicing. A: Effect of missense mutations on natural acceptor and donor splicing sites. Analysis of the splice-sites revealed 3 possible splice-site abolishments. B: Effect of missense mutations on cryptic and novel acceptor and donor sites. The analysis suggested possible changes in 3 sites involving the activation of cryptic and novel donor/acceptor sites. Note: the range of values is given in parenthesis for each splicing algorithm. (DOC) [file pgen.1003632.s007.doc]

| cDNA change | AA change | MaxEnt | NNSPLICE | HSF | Comment |
| --- | --- | --- | --- | --- | --- |
| c194G>T | *p.S65I* | -100% | -99% | -14.10% | Predicted change at donor site 1 bps downstream: -71.0% |
| c548G>T | *p.G183V* | -18.80% | -6.50% | -4.60% | Predicted change at acceptor site 1 bps upstream: -9.9% |
| c.638A>T | *p.K213M* | 16.70% | -4.90% | -5.00% | Predicted change at donor site 2 bps downstream: -8.9% |

Supplementary Table S5A

| cDNA change | AA change | MaxEnt  (0-12) | NNSPLICE  (0-1) | HSF  (0-100) | Comment |
| --- | --- | --- | --- | --- | --- |
| c358C>G | *p.L120V* | 0 ⇒ 6.52 | 0 ⇒ 0.45 | 0 ⇒ 79.65 | New donor site may be activated |
| c1025G>T | *p.R342L* | 0 ⇒ 1.93 | 0 ⇒ 0.43 | 87.37 ⇒ 90.53 (+3.6%) | Cryptic acceptor site may be activated |
| c1115T>C | *p.L372P* | 4.20 ⇒ 6.23 (+48.5%) | 0 ⇒ 0.71 | 79.06 ⇒ 81.04 (+2.5%) | Cryptic donor site may be activated |

Supplementary Table S5B
