# Supplementary material for: Functional Characterisation of Alpha-Galactosidase A Mutations as a Basis for a New Classification System in Fabry Disease
Source: PLoS Genet. 2013 Aug 1;9(8):e1003632. doi: 10.1371/journal.pgen.1003632 (PMC3731228; doi:10.1371/journal.pgen.1003632)
Supplement: Text S1 — Additional method. Description of the method applied to reveal alterations of mRNA splicing. (DOC) [file pgen.1003632.s008.doc]

**Text S1:**

**Additional method**

**Splicing analysis**

The splicing prediction was performed using Alamut 2.2 with MaxEntScan [42], NNPLICE [43] and Human Splicing Finder (HSF) [44] modules. Average changes detected by all three algorithms at acceptor and donor sites exceeding 5% as compared to the wild type sequence were considered “interesting” and are listed in Supplementary Table 4A for changes affecting natural sites and in Supplementary Table 4B for changes leading to activation of cryptic and novel sites.

Additional References:

42. Yeo G, Burge CB (2004) Maximum entropy modeling of short sequence motifs with applications to RNA splicing signals. J Comput Biol. 11(2-3):377-94.

43. Reese MG, Eeckman FH, Kulp D, Haussler D (1997) Improved splice site detection in Genie. J Comput Biol. 4(3):311-23.

44. Desmet FO, Hamroun D, Lalande M, Collod-Béroud G, Claustres M et al. (2009) Human Splicing Finder: an online bioinformatics tool to predict splicing signals. Nucleic Acids Res. 37(9):e67.
